# Heterotypic 3D Model of Breast Cancer Based on Tumor, Stromal and Endothelial Cells: Cytokines Interaction in the Tumor Microenvironment

**DOI:** 10.3390/cells15020145

**Published:** 2026-01-14

**Authors:** Anastasia Leonteva, Alina Kazakova, Ekaterina Berezutskaya, Anna Ilyina, David Sergeevichev, Sergey Vladimirov, Maria Bogachek, Igor Vakhrushev, Pavel Makarevich, Vladimir Richter, Anna Nushtaeva

**Affiliations:** 1 Scientific Center of Genetics and Life Sciences, Sirius University of Science and Technology, 1 Olimpiysky Avenue, Krasnodar Region, Sirius 354340, Russia; anastleont@mail.ru (A.L.); kazakova.ala@talantiuspeh.ru (A.K.); ilina.ana@talantiuspeh.ru (A.I.); sergeevichev.ds@talantiuspeh.ru (D.S.); vladimirov.sk@talantiuspeh.ru (S.V.); maryambogachek@gmail.com (M.B.); 2 Institute of Chemical Biology and Fundamental Medicine Siberian Branch of the Russian Academy of Sciences, Akad. Lavrentiev Ave. 8, Novosibirsk 630090, Russia; kaitha@berezus.ru (E.B.); richter@1bio.ru (V.R.); 3 Department of Natural Sciences, Novosibirsk State University, Pirogova Str. 2, Novosibirsk 630090, Russia; 4 Laboratory of Cell Biology, Institute of Biomedical Chemistry, Pogodinskaya Str., 10, Moscow 119121, Russia; vakhrunya@gmail.com; 5 Centre for Regenerative Medicine, Lomonosov Moscow State University, Lomonosovskiy Ave., 27, b.10, Moscow 119192, Russia; makarevichpi@my.msu.ru; 6 Institute of Experimental Cardiology, E.I. Chazov National Research Medical Centre of Cardiology, Akademika Chazova St., 15, Moscow 121552, Russia

**Keywords:** tumor microenvironment, breast cancer, cancer-associated fibroblast, endothelial cells, spheroid, heterotypic, cytokines, SDF1, VEGF, STRING

## Abstract

The recreation of the tumor microenvironment remains a significant challenge in the development of experimental cancer models. The present study constitutes an investigation into the interconnection between tumor, endothelial and stromal cells in heterotypic breast cancer spheroids. The generation of models was achieved through the utilization of MCF7, MDA-MB-231, and SK-BR-3 tumor cell lines, in conjunction with endothelial TIME-RFP cells and either cancer-associated (BrC4f) or normal (BN120f) fibroblasts, within ultra-low attachment plates. It was established that stromal cells, most notably fibroblasts, were conducive to the aggregation of tumor cells into spheroids and the formation of pseudovessels in close proximity to fibroblast bands. In contrast to the more aggressive tumor models MDA-MB-231 and SK-BR-3, microenvironment cells do not influence the migration ability of MCF7 tumor cells. Heterotypic spheroids incorporating CAFs demonstrated a more aggressive and immunosuppressive phenotype. Multiplex immunoassay analysis of cytokines, followed by STRING cluster analysis, was used to identify key processes including angiogenesis, invasion, stem cell maintenance, and immunosuppression. Furthermore, a cluster of cytokines (LIF, SDF-1, HGF, SCGFb) was identified as potentially involved in the regulation of PD-L1 expression by tumor cells. This finding reveals a potential mechanism of immune evasion and suggests new avenues for therapeutic investigation.

## 1. Introduction

The tumor microenvironment (TME) of breast cancer (BC) plays a crucial role in tumor progression, immune evasion and resistance to conventional anti-cancer therapy [1]. The TME of BC is characterized by high plasticity, undergoing continuous changes and stage-specific adaptations in response to a multitude of intrinsic and extrinsic factors associated with tumor cells [2]. These TME changes are characterized by disrupted cytokine and growth factor networks, disrupted signaling pathways and altered molecular signatures in the stroma [3]. A pivotal element of the TME research pertains to the analysis of cell-to-cell interactions. It is evident that tumor, stromal and endothelial cells engage in a dynamic interplay through the secretion of cytokines or exosome, thereby inducing alterations in phenotypes, vasculogenesis and cancer progression [4]. Tumors are characterized by a rigid extracellular matrix (ECM), which is a non-cellular component of the TME and distinguishes them from normal tissue [5]. Many cell types respond to matrix stiffness by change their phenotypes. Cancer-associated fibroblasts (CAFs) have been identified as critical to the process of tissue stiffening in tumors. It is well established that normal fibroblasts generate a matrix that is softer than that produced by CAFs [6]. It is also evident that cells within tumors, including cancerous and stromal cells, respond to matrix stiffness, thereby modulating their phenotypes and regulating cancer progression [7]. Key factors influencing cancer development include matrix stiffness, drug resistance, cancer stem cell properties, angiogenesis and immune responses. It is imperative that in vitro tumor models are utilized for the regulated examination of novel anti-cancer therapies and for elucidating the cellular and molecular mechanisms of tumorigenesis [8].

For a period exceeding five decades, BC cell lines have been instrumental in the research of prognoses, protein biomarkers, morphological differences, and genetic mutations. The application of a categorization system to BC cell lines enables the distinction between Luminal A, Luminal B, Luminal-HER2, HER2+, and triple-negative breast cancer (TNBC). This enhances the precision of analyses and experimental designs, facilitating novel advancements in the field of scientific research [9]. The intricate interrelationships that characterize the in vivo context are lost when cell lines are cultivated on plastic in two dimensions (2D). However, 2D culture remains the most prevalent approach for in vitro studies in BC research [10]. In 2D cell cultures, the important aspects of tumor biology, such as interactions between different cell types and between cells and the ECM, are typically ignored [11].

Three-dimensional (3D) models in vitro that are able to replicate the TME are experiencing a rise in application in the field of tumor research [12]. Spheroid models are considered to be the elementary and most reproducible 3D models [12]. As demonstrated by these models, the topography, metabolism, signaling and gene expression levels of the models are very similar to those of tumor cells in solid tumors in vivo [13]. These properties greatly increase the range of applications for studying human tissue physiology and clarifying the pathophysiology of cancer [14,15,16]. Although more complex in vivo models (i.e., animal models) are still necessary in the final stages of preclinical research, they pose significant challenges, including low efficiency, ethical issues, much longer experiment times, and higher costs. Against this backdrop, preclinical 3D models have been the subject of active study in recent years [16]. The underlying cancer models in each category—in vitro, in vivo, ex vivo and in silico—are undergoing a renaissance, accelerating the pace at which new knowledge is acquired, from laboratory research to clinical application [17]. The incorporation of components from the TME, in conjunction with the presence of interacting cell types, has the potential to enhance the significance of spheroid models in the domains of translational and intercellular interaction research.

A recent global study demonstrated that, although over 80% of researchers recognize the importance of 3D models, the majority do not utilize them regularly in their research, primarily due to their high cost [18]. The establishment of standardized and reproducible protocols for generating in vitro tumor models with consistent size, morphology, and architecture is critical for several key objectives in biomedical research. Firstly, such standardization facilitates the broader adoption of these models across laboratories, ensuring comparability of results. Secondly, it enhances the predictive value and clinical translatability of preclinical findings. Finally, robust in vitro models directly support the ethical principles of the 3Rs (Replacement, Reduction, and Refinement) by providing a viable alternative to animal studies, thereby aligning research practices with contemporary animal welfare standards [16,18]. Notwithstanding considerable advances, the development of spheroid models continues to encounter significant technical challenges. A thorough review of the extant literature indicates that the efficacy of the methodologies reported varies significantly. The performance of these methodologies is found to be contingent upon the specific cell type, the particular culture conditions, and the intended application. Consequently, the selection and optimization of a spheroid generation protocol remain non-trivial tasks that significantly impact the biological relevance and experimental reproducibility of the resulting model [19,20]. In our previous work, we proposed uniform conditions for the 3D cultivation of three breast cancer cell lines (MCF7, MDA-MB-231, and SK-BR-3) that allow the formation of proliferating spheroids. The addition of fibroblasts to the culture medium has been demonstrated to expedite the formation of tumor-stromal spheroids [21]. The findings of this study demonstrated that 17-β estradiol (E2) stimulation led to the proliferation of tumor cells in both 3D and heterotypic spheroids (3D-2) consisting of tumor and stromal cells, irrespective of the type of BC cells employed in the model. Conversely, the 2D model demonstrated resistance to E2 in MDA-MB-231 cells. In 3D models, the MDA-MB-231 cells exhibited a loss in sensitivity to the proliferative effect of TGF-β, while the SK-BR-3 cells demonstrated an increase in sensitivity. As demonstrated in the extant literature, 3D and 3D-2 cellular models of BC constitute a significant instrument in the study of tumor progression and the evaluation of novel antitumor approaches, notwithstanding the prevalence of 2D models. The presence of CAFs within the model has been shown to induce alterations in the expression levels of MICA/B and PD-L1 by tumor cells within the 3D-2 model [22]. The subsequent phase in the progression of heterotypic 3D models as a methodology for the examination of intercellular interactions and tumor formation mechanisms involves the formation of heterotypic spheroids consisting of tumor cells, fibroblasts and/or endothelial and/or immune cells. These spheroids can be utilized to create in vitro models that resemble the TME of tumors in vivo [4,22]. The augmentation of the biological characteristics of spheroid models will result in a substantial enhancement of their value and translational potential.

Cytokines and chemokines, produced by various components of the TME, have been shown to play a pivotal role in regulating tumor initiation, progression, and metastasis [23]. It has been demonstrated by preceding studies that the BC microenvironment is distinguished by the activation of signaling cascades involving the interaction of multiple cytokines and chemokines [24,25]. In this study, the applicability and reproducibility of in vivo features were assessed by characterizing the developed heterotypic spheroid models comprehensively, including the secretion of cytokines and chemokines.

In this context, these studies focused on developing a simple, cost-effective method adapted from the liquid overlay technique for generating spheroids that could be used in any standard cell culture laboratory, regardless of whether or not the laboratory has specialized equipment or advanced technical expertise. The objective of this study was to develop a heterotypic 3D cell model of BC and to verify the functional characteristics and molecular profile of model, with the aim of replicating the TME in vivo. The selection of tumor cell models was based on the hormonal status of the cell lines: the hormone-positive MCF-7, the HER2-positive SK-BR-3, and the triple-negative MDA-MB-231. Two different types of patient-derived cultured fibroblasts originating from normal or tumor breast tissue of the patient were used to create spheroid models. The cell culture of BrC4f was categorized as CAFs, while the BN120f cell culture represented normal fibroblasts [22,26]. TIME-RFP cells are microvascular endothelial-like cells. In order to confirm the formation of an immunosuppressive tumor microenvironment, multiplex screening was performed using a human 48-plex cytokine panel. Depending on whether normal fibroblasts or CAFs were used in a given 3D model, the following points were evaluated in the present study: (i) the formation features and characterization of the heterotypic spheroid model; (ii) analysis of dynamics in cell adhesion; (iii) migration/invasive properties of cells depending on the type of matrix; (iv) analysis of alterations in cytokine secretion and their relationship with PD-L1.

## 2. Materials and Methods

### 2.1. Cell Lines

MCF-7 (#ACC 115, DSMZ, Braunschweig, Germany), MDA-MB-231 (#ACC 65, DSMZ, Braunschweig, Germany) and SK-BR-3 (ATCC, #HTB-30, Manassas, VA, USA) were purchased from the American Type Culture Collection (ATCC, Manassas, VA, USA). BrC4f and BN120f were obtained in the Laboratory of Biotechnology in the Institute of Chemical Biology and Fundamental Medicine SB RAS (ICBFM, Novosibirsk, Russia). TIME-RFP cell line was kindly provided by I. V. Vakhrushev from the Institute of Biomedical Chemistry (IBMC, Moscow, Russia). RT-PCR analysis revealed the absence of mycoplasma contamination in all cell lines. The genetic identification of cell lines was performed using the GOrDIS Plus kit (GORDIZ, Moscow, Russia). The STR profiles correspond to those published in international databases, namely ATCC, DSMZ and Cellosaurus.

MCF-7, SK-BR-3 and MDA-MB-231 cell lines were cultivated in Dulbecco’s Modified Eagle Medium/Nutrient Mixture F-12 (DMEM/F12) (#42400028, Gibco™, New York, NY, USA). BrC4f and BN120f cells were cultivated in Iscove’s Modified Dulbecco’s Medium (IMDM) (#I7633-10X1L, Sigma-Aldrich, St. Louis, MO, USA) [27,28]. All cultures’ media were supplemented with 10% fetal bovine serum (FBS) (#A316040, Thermo Fisher, Waltham, MA, USA) and with 250 mg/mL amphotericin B and 100 U/mL penicillin/streptomycin (#15140122, Gibco™, Waltham, MA, USA). TIME-RFP cells were cultivated in EndoLife medium (Appscience, Moscow, Russia) supplemented with 10% of FBS. Cells were cultured at 37 °C with 5% CO_2_. For subculturing, adherent cells were washed with 2 mL of PBS and detached by incubation with 400 µL of TrypLE™ (#12604013, Gibco, Invitrogen) for 3–5 min at 37 °C. Following detachment, the cells were resuspended in 1 mL of culture medium. A proportion of the suspension was subsequently transferred to a new culture flask containing 5 mL of fresh medium.

### 2.2. Spheroids Formation

Forced floating methods are partly based on the use of plastic surfaces treated to prevent the attachment of cells to these surfaces and to promote their aggregation. Thus, so-called “ultralow attachment” (ULA) multiwell microplates are marketed [29]. The homo- and heterotypic 3D-models of breast cancer were established using the liquid overlay technique in ULA 96-well or 24-well Nunclon™ Sphera™ U-shaped-bottom plates (#174925 and #174930 Thermo Scientific, Waltham, MA, USA) [22,30]. Homotypic models consisted of tumor cells (3De), while heterotypic models consisted of tumor cells and endothelial cells (3D-2) or tumor, endothelial and stromal cells (3D-3).

Cells were washed with 2 mL of 1X PBS and incubated at 37 °C for 3–5 min with 400 μL Accutase Cell Detachment Solution (ACC-1B, Capricorn Scientific, Ebsdorfergrund, Germany). After detachment, cells were washed with 1 mL of culture medium and counted using a LUNA-II^TM^ cell counter (Logos Biosystems, Anyang-si, Republic of Korea). After centrifugation (5 min, 330 rcf), the cells were suspended in 200 µL of DMEM/F12 containing 1X GlutaMAX™ Supplement (35050061, Gibco™, New York, NY, USA), 1X Antibiotic-Antimycotic (15240062, Gibco™, New York, NY, USA), 20 ng/mL EGF (Epidermal Growth Factor; E9644, Sigma-Aldrich, Burlington, MA, USA), 20 ng/mL fibroblast growth factor basic (bFGF, PHG0261, Gibco™, New York, NY, USA), 5 µg/mL insulin (I9278, Sigma-Aldrich, Burlington, MA, USA), 2% B27 Plus Supplement (A35828010, Gibco™, Burlington, MA, USA), and 4% Albumin Bovine Serum Fraction V (BSA, 126593, Sigma-Aldrich, Burlington, MA, USA). To construct heterotypic models, tumor cells were suspended with fibroblasts to reach a tumor/endothelial cell ratio of 1:2 for 3D-2 models or tumor/endothelial/stromal cell ratio of 1:2:4 for 3D-3 models, and all cell types were seeded simultaneously with total cell number of 2500 cells/well for 96-well plates or 200,000 cells/well for 24-well plates. Agarose hydrogel (2%, 50 µL) was added to each well of a 96-well culture plate (TPP, Trasadingen, Switzerland) and incubated at 37 °C for 1 h. Stromal cells were seeded in 100 µL of growth medium at a concentration of 2500 cells per well. The plates were then subjected to an additional 72-h incubation at 37 °C, a process which was intended to facilitate the formation of stromal spheroids (3Df) within the culture.

Three immortalized BC lines were used as tumor cells for spheroid formation: ESR1-positive breast adenocarcinoma MCF-7; HER2-positive breast adenocarcinoma SKBR-3; triple-negative breast adenocarcinoma MDA-MB-231. Tumor cells were co-cultured with stromal cells (cancer-associated fibroblasts BrC4f or normal fibroblasts BN120f) and/or endothelial cells TIME-RFP.

The preparation of cell spheroids for the subsequent studies was undertaken in accordance with this methodology.

### 2.3. Confocal Microscopy

The localization of cells within spheroids was determined by means of vital cell staining with fluorescent dyes. Fibroblast cell lines were stained with CellTracker Green (Invitrogen, Carlsbad, CA, USA), and tumor cells were stained with CytoTracer Blue CMAC (4682-10 mg, Lumiprobe, Moscow, Russia). On the 3rd day, spheroids were washed with PBS, transferred to a flat-bottomed plate (Eppendorf, Hamburg, Germany), and visualized using an L-710 (Carl Zeiss, Oberkochen, Germany) confocal microscope. Images were analyzed using the Fiji software (ImageJ 2.16.0/1/54p, Java 1.8.0_442 (64-bit)).

### 2.4. Time-Lapse of Spheroid Formation Process

The CELENA X High Content Imaging System (Logos Biosystems, Anyang-si, Republic of Korea) was utilized to analyze the process of spheroid formation. As outlined in Section 2.2, it is necessary to adhere to the established protocol when conducting the seeding of tumor, endothelial and stromal cells. Subsequent to this, the plate was positioned within a CELENA X incubation chamber, and time-lapse imaging of each well was initiated. The imaging time interval was 15 min. Three independent experiments were performed. Subsequent to the conclusion of the experiment, the photographic materials were amalgamated into a video file.

### 2.5. Live/Dead Staining

Spheroids obtained previously were subjected to staining with 1 µg/mL fluorescein diacetate (#F1303, Thermo Fisher, USA) diluted in DMEM/F12 FBS-free medium for a period of 45 min at a temperature of 37 °C. Following the washing of the FDA with 1X PBS, the spheroids were stained with 5 µg/mL propidium iodide (PI) (BD Bio-sciences, NJ, USA) and 1:1000 Hoechst 33342 (Invitrogen, USA) diluted in PBS for 10 min at 37 °C. Three independent experiments were performed (N = 3). The results were then subjected to visualization using a Nikon Eclipse Ti-S series fluorescence inverted microscope. Image analysis was conducted utilizing the NIS-Elements software (Nikon Instruments Inc., version 5.30.05 64-bit, Melville, NY, USA).

### 2.6. Reattachment Test

The migration capacity of cells within the spheroids was monitored in real-time with the xCELLigence Real Time Cell Analyzer (RTCA) system (ASEA Biosciences) by measuring cell-to-electrode responses on E-plates with the integrated microelectronic sensor arrays (ACEA Biosciences Inc., San Diego, CA, USA). The seeding of tumor, endothelial and stromal cells must be conducted in strict accordance with the established protocol, delineated in Section 2.2. Following a 72-h cultivation period, the cells are transferred to E-plate wells in a total volume of 100 µL of DMEM/F12 medium containing 10% FBS, thus providing the cells with adhesion components. In standard E-Plates, the gold biosensors are integrated bottomed wells from polyethyleneterephthalate (PET) plates. 3D-models attached to the bottom of E-plates and cell migration was monitored in real time for 96 h. The cell index (CI) was calculated for each E-plate well by RTCA Software 1.2 (Roche Diagnosis, Meylan, France) using 16-well E-plates with integrated microelectronic sensor arrays (ACEA Biosciences Inc., San Diego, CA, USA).

### 2.7. Investigation of the Potential of Cells in Spheroids for Invasion and Migration

The wells of a 96-well plate (TPP Techno Plastic Products AG, Trasadingen, Switzerland) have been coated with a substrate of either Matrigel™ hydrogel (BD Biosciences, San Jose, CA, USA) or cold-water fish skin gelatin (Sigma, Oakville, ON, Canada). The hydrogel was polymerized by incubating the plate at 37.0 ± 1.0 °C for 30 min. Thereafter, the prepared spheroids were transferred onto the hydrogel-coated substrate for further culture under standard conditions. The results were then visualized using a Nikon Eclipse Ti-S series fluorescence inverted microscope. Image analysis was conducted utilizing the NIS-Elements software (Nikon Instruments Inc., version 5.30.05 64-bit, Melville, NY, USA). A thorough analysis was conducted on the outcomes of three independent experiments.

### 2.8. Flow Cytometry

All analyses were performed using a FACS Canto II flow cytometer (BD Biosciences, Franklin Lakes, NJ, USA), and the data were analyzed using FACSDiva Software Version 6.1.3. (BD Biosciences). The formation of spheroids is to be conducted in accord with the established protocol, as delineated in Section 2.2. The initial gating of cells was based on a comparison of forward scatter and side scatter in order to eliminate the presence of minor particulate matter. This process resulted in the collection of 10,000 events from the specified population. The following antibodies were utilized in the analysis: anti-PD-L1-APC (#RT2568050) from Sony and anti-CD44-APC and anti-CD24-PECy7 from BD Pharmigen (Franklin Lakes, NJ, USA) (#560890 and #561646, respectively). The spheroids were collected, washed with 1× PBS on two occasions, and then dissociated with 500 µL of Accutase^®^ (Capricorn, Ebsdorfergrund, Germany) for a period of 10 min at 37 °C in 5% CO_2_, unless otherwise indicated. In order to facilitate the mechanical enzymatic dissociation of spheroids, the suspension was subjected to 10 cycles of up-and-down pipetting, thereby inducing shear forces. The addition of serum supplement media and subsequent centrifugation at 1000 rpm for 5 min was employed to neutralize Accutase^®^. Finally, the cell suspension was placed on a pre-separation filter with a pore size of 70 µm (BD Biosciences, NJ, USA), which was filled with 800 µL of PBS with 2% FBS, and then subjected to centrifugation at 1000 rpm for 5 min. Following the process of cell enrichment, the cells were subjected to staining with a combination of antibodies and subsequently analyzed via flow cytometry. The NucBlue™ Live ReadyProbes™ (Invitrogen™, Waltham, MA, USA) were added to the cell suspension immediately prior to detection via a flow cytometer. In addition to determining the cell number, the LUNA FL Cell Counter was utilized to quantify the number of single cells in order to monitor complete dissociation of the spheroids.

### 2.9. Western Blot

The procedure for the formation of spheroids is to be conducted in line with the established protocol, as set out in Section 2.2. After 5 days of cultivation of spheroid, the spheroids from each well were transferred to centrifuge tubes. Subsequently, the samples were subjected to a centrifugal process at a speed of 1000× *g* for a duration of 15 min at a temperature of 4 °C. The sediment of spheroids was lysed in RIPA buffer (150 mM NaCl, 1% Triton X-100, 0.1% SDS, 50 mM Tris-HCl pH 7.4) containing 1 × protease inhibitor cocktail (ServiceBio, Wuhan, China). Protein from spheroid lysates was quantified with ProteOrange Protein Quantification Kit (Lumiprobe, Moscow, Russia) or Bradford Protein Assay Kit (FineTest, Wuhan, China). A protein from spheroid lysates and 4 × sample loading buffer (Biolabmix, Novosibirsk, Russia) was loaded per well into a 4–20% SDS PAGE gel (WSHT, Shanghai, China). Proteins were wet transferred to PVDF or nitrocellulose membranes (Bio-Rad, Hercules, CA, USA), blocked for 1 h in 5% nonfat milk, and incubated overnight at 4 °C and constant shaking with primary antibodies: fibronectin (1:2000, ABclonal, Wuhan, China), vimentin (1:5000, ABclonal, Wuhan, China) and GAPDH (1:20,000, ABclonal, Wuhan, China). Membranes were washed 5 times in Tris-buffered saline containing 0.1% Tween-20 (TBST) for 30 min and incubated for 1 h at room temperature and constant shaking with HRP-conjugated anti-rabbit antibodies (1:10,000, ABclonal, Wuhan, China). Membranes were washed 5 times in TBST, developed with enhanced chemiluminescence (ServiceBio, Wuhan Optics Valley, China), and visualized with the ChemiDoc Imaging System (Bio-Rad, Hercules, CA, USA). Images were acquired with Image Lab Software (Bio-Rad, Hercules, CA, USA).

### 2.10. Immunocytochemistry

Cells (1 × 10^4^) were cultivated in four-well culture slides (BD Falcon, Bedford, MA, USA) and subsequently washed with PBS and fixed in 10% Neutral-Buffered Formalin. In order to block non-specific antibody binding, the cells were incubated in 1% BSA (Sigma-Aldrich, Burlington, MA, USA) and 0.3 M glycine in PBST buffer (PBS with 0.1% Tween 20) for a period of 30 min at RT. Then, the cells were exposed to anti-fibronectin (A12977, ABclonal, Wuhan, China) antibodies for a duration of 60 min at RT. For the purpose of visualization, Alexa Fluor 555-conjugated (#A32727, Invitrogen, Waltham, MA, USA) secondary antibodies were utilized for a period of one hour at room temperature. The results were then visualized using a Nikon Eclipse Ti-S series fluorescence inverted microscope. Image analysis was conducted utilizing the NIS-Elements software (Nikon Instruments Inc., version 5.30.05 64-bit, Melville, NY, USA).

### 2.11. xMAP Analysis

To measure the levels of cytokines and chemokines in spheroids the xMAP analysis using Bio-Plex Pro Human Cytokine Screening 48-Plex Panel (#12007283, Bio-Rad Laboratories, Hercules, CA, USA) and QuattroPlex Lab (Dia-M, Moscow, Russia) was conducted. The procedure for the formation of spheroids is to be conducted in line with the established protocol, as set out in Section 2.2. To obtain 3D-models for analysis 200,000 cells/well were seeded on 24-well low-attachment plates and after 5 days of cultivation spheroids with medium from every well were transferred to centrifuge tubes, BSA was added to a final concentration of 0.5%, and the samples were centrifuged at 1000× *g* for 15 min at 4 °C to remove spheroid and the cell debris. The culture supernatants were collected, filtered through a 0.22 μm filter (TPP Techno Plastic Products AG, Trasadingen, Switzerland) and stored in aliquots at −80 °C. Aliquots of the supernatant were then added to the assay plate. The remaining analysis steps were performed according to the kit manufacturer’s instructions.

### 2.12. Statistical Analysis

A statistical analysis was performed on the results of three independent experiments. The results were analyzed using GraphPad Prism v.9.0 (GraphPad Software, San Diego, CA, USA) and two-way Analysis of Variance (ANOVA). Tukey’s HSD and non-parametric Mann–Whitney U-test were performed. The results obtained are presented as the mean values of three repetitions, with their respective standard errors.

## 3. Results

### 3.1. The Formation of a Heterotypic 3D Cell Model of Breast Cancer

**Figure 1:**
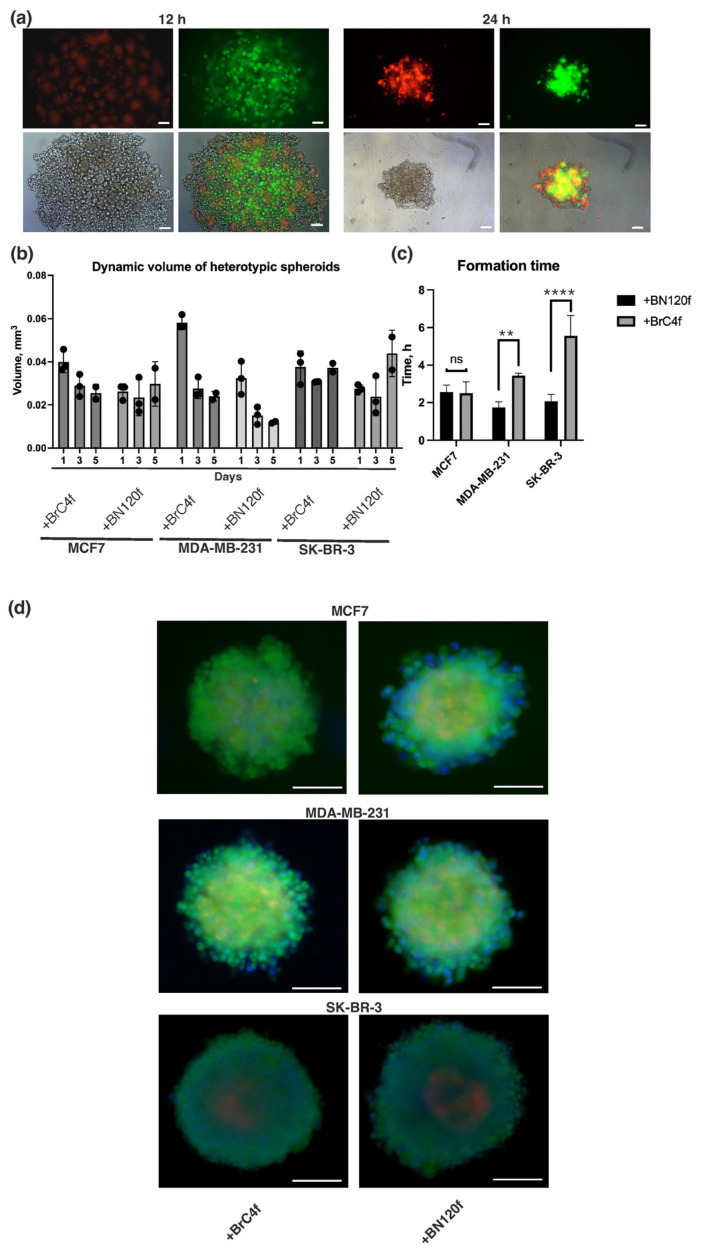
The characteristics of formation and growth of heterotypic spheroid models of breast cancer. (**a**) The process of adhesion between other cells in a spheroid of a homotypic nature using the MCF7 cell line as an example. Fluorescence microscopy was utilized, with red signal indicating endothelial cells and green signal indicating stromal cells. The scale bar is 100 μm. (**b**) Dynamics of changes in spheroid volume over 5 days. (**c**) Time of formation and compaction of spheroids according to time-lapse photography in transmitted light. ns—not significant; ** *p* < 0.01; **** *p* < 0.0001 determined by unpaired two-tailed Student’s *t* test. (**d**) Analysis of cell viability in a spheroid on day 5 of co-culture. Green signal (FDA)—live cells, red (PI)—dead cells, and endothelial cells (RFP), blue (Hoechst 33342)—total number of cells. The scale bar is 100 μm.

The dynamic interactions between cancer cells and cells in the TME have been demonstrated to influence tumor progression, metastasis, and patient prognosis [1]. Previously, the conditions for the formation and culture of heterotypic spheroid models consisting of tumor cells, such as MCF7, SK-BR-3, and MDA-MB-231, and stromal cells, such as cancer-associated fibroblasts (BrC4f) and normal fibroblasts (BN120f), were developed [22,26]. The approach to forming heterotypic spheroid models (3D-3) is based on the direct mixing of tumor cells with other cells from the microenvironment in the desired ratios (tumor:stromal:endothelial cells, 1:4:2, respectively). In the present study, the previously obtained heterotypic spheroid model was supplemented with endothelial cells (TIME-RFP) under preselected conditions [21,22]. As a control, homotypic tumor spheroids (3D) and heterotypic spheroids (3D-2, tumor-endothelial cells) were cultured (Figure S1). During the initial hours of culturing, various cell types aggregated with each other through the mechanism of homotypic adhesion, resulting in the formation of distinct compact homotypic clusters. These clusters then underwent compaction, ultimately giving rise to a spheroid. The inner core of the spheroid was formed by stromal cells, which were surrounded by tumor and endothelial cells (Figure 1a). Stromal, but not endothelial, cells have been demonstrated to contribute to the formation and compaction of heterotypic spheroid models (Figure 1b, Figures S1 and S2). The incorporation of normal BN120f fibroblasts into the model led to a more rapid development of spheroids in comparison to CAFs BrC4f, a finding that is in accordance with previously reported data (Figure 1c) [22]. Following a five-day culture period, the spheroids were predominantly composed of viable cells, with a minor presence of dead cells (Figure 1d and Figure S2).

**Figure 2:**
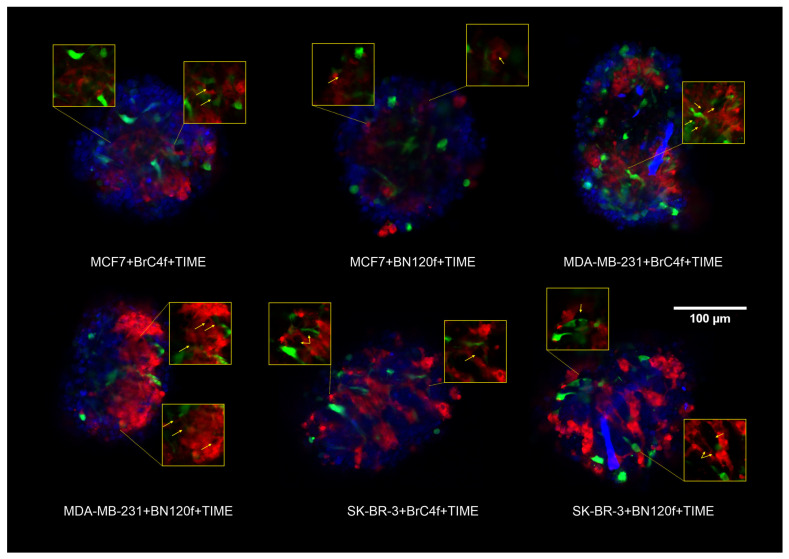
The structure and internal organization of heterotypic spheroid of breast cancer. The phenomenon under consideration is characterized by the presence of an intricate network of vessel-like structures, arranged in a web-like configuration, within the framework of spheroidal models. The yellow frame with an arrow shows the formation of a structure that looks the lumen-like of a vessel. The scale bar is 100 µm. Blue signal—tumor cells, green signal—stromal cells, red signal—endothelial cells.

Crosstalk between tumor and endothelial cells (ECs) has been shown to drive a critical process of new blood vessel formation known as angiogenesis, which is a hallmark of tumorigenesis [31]. No discrepancy in endothelial cell localization was observed depending on the type of added fibroblasts; however, differences were observed depending on the tumor cell type (Figure 2). Confocal microscopy analysis revealed that in spheroids from triple-negative BC MDA-MB-231 cells, ECs were organized into clusters, forming multiple poles at the periphery of the model (Figure 2 and Figure S2). For 3D models from MCF7 and SK-BR-3 cells, ECs demonstrated a tendency to aggregate into distinct capillary-like structures (Figure 2 and Figure S2). It is important to note that stromal cells were located in close proximity to the strands of endothelial cells. The addition of 2.5% Matrigel has been demonstrated to enhance the formation and compaction of spheroids. However, rather than supporting organized pseudovessel formation, it triggered increased invasion and disordered surface spreading of endothelial cells (Figure S2c).

Thus, co-cultivation of different cells in a heterotypic model has been shown to stimulate the formation of vessel-like structures without the need for the addition of vascular endothelial growth factor (VEGF) or other angiogenesis inducers to the culture medium, provided that the selected cell ratios are correct.

### 3.2. Reattachment Test of Spheroid Models in Real-Time System

The application of the xCELLigence RTCA system facilitates the analysis of cell adhesion and proliferation cells in real time [26]. Spheroids after five-days cultivation in serum-free medium were transferred to the E-plate well of the xCELLigence RTCA cell analyzer in a culture medium containing 10% fetal bovine serum (FBS). Subsequent to the application of the E-plate, the plate was placed in an incubator at 37 °C in a humidified atmosphere of 5.0 ± 0.5% CO_2_ for 30 min, with the purpose of allowing the spheroids to settle naturally to the bottom of the well.

**Figure 3:**
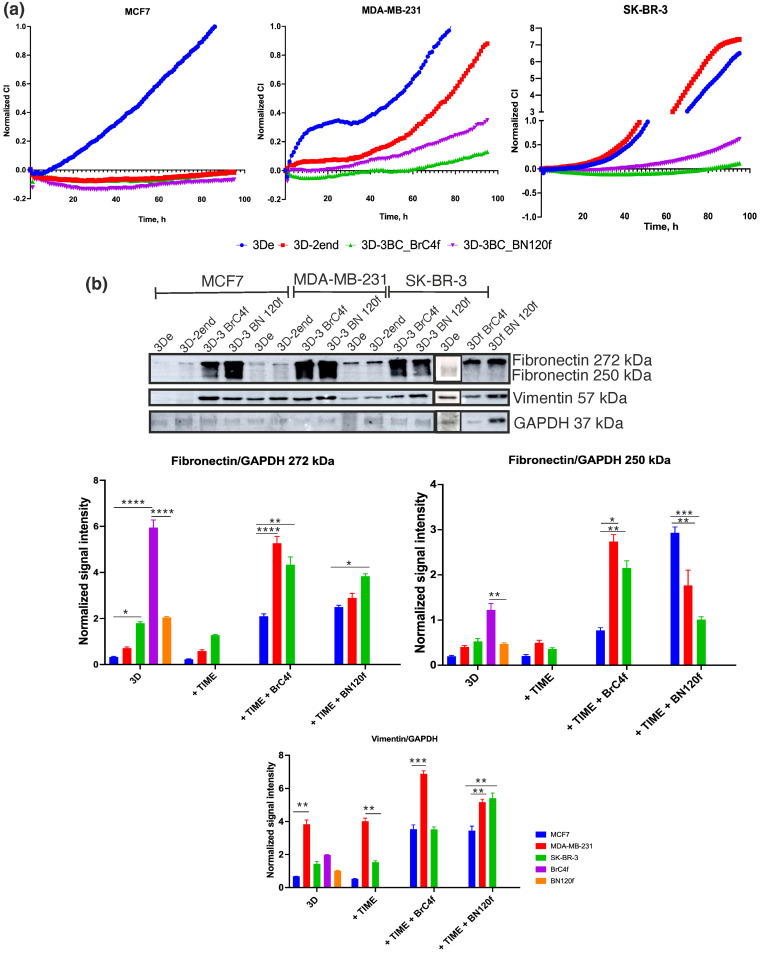
Migration potential of a heterotypic spheroid model of breast cancer (3D-3). (**a**) The reattachment test for a spheroid in xCELLigence analyzer (typical data from real-time breast tumor cell reattachment and proliferation curves). (**b**) The immunoblotting analyses of fibronectin and vimentin in spheroids. One representative Western blot of two independent experiments is shown. Quantification of the protein expression was normalized to GAPDH as a loading control. The protein bands of target were quantified as relative values to loading control bands. The level of fibronectin protein, such as 272 kDa and 250 kDa, in homotypic and heterotypic spheroids. The difference between the experimental groups compare with control (homotypic spheroid) was statistically significant at * *p* < 0.05; ** *p* < 0.01; *** *p* < 0.001; **** *p* < 0.0001 (non-parametric Mann–Whitney U-test).

Typical curves of cell “reattachment” and proliferation demonstrate that different molecular subtypes of models of BC have different adhesive and invasive properties (Figure 3a, blue curve). A rapid increase in the CI for 3De MCF7 cells is indicative of high cell adhesive capacity, while MDA-MB-231 and SK-BR-3 cells are characterized by a delay in the “reattachment” process of spheroid’s cell, followed by rapid cell exit from the model and a high rate of cell proliferation. The incorporation of TIME endothelial cells (red curve) or stromal cells (purple and green curves) into the MCF7 spheroid model has been shown to promote the formation of denser and more organized spheroids, thereby inhibiting the invasive and migratory potential of tumor cells, as reflected in a consistently negative CI (Figure 3a). The dense ECM produced by the stroma may create a physical barrier that limits the motility and spread of MCF7 cells, which do not initially have a high invasive potential [32]. Conversely, for spheroids derived from MDA-MB-231 and SK-BR-3 cells, the incorporation of TIME endothelial cells (red curve) within the initial 40 h impedes the “reattachment” process of spheroid cells, resulting in expeditious cell egress from the model and a substantial rate of cell proliferation (Figure 3a). The incorporation of stromal cells into 3D of MDA-MB-231 and SK-BR-3 cells has been demonstrated to encourage the development of more compact models. This is evidenced by the observation of a negative CI at 40 h for MDA-MB-231 cells and 60 h for SK-BR-3 cells, subsequently followed by cell adhesion and proliferation. Heterotypic spheroids formed in conjunction with CAFs BrC4f fibroblasts have been found to be the densest of all spheroid types.

**Figure 4:**
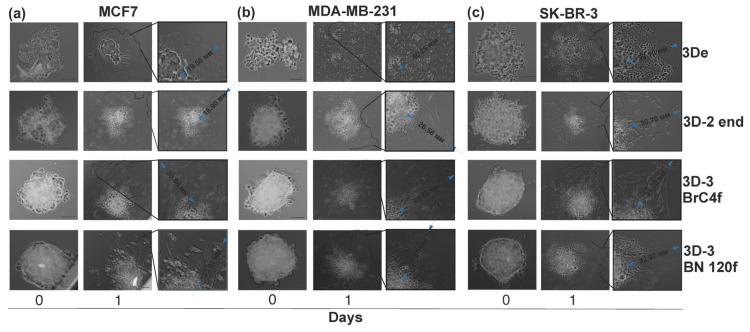
Migration cell from 3D cellular models of BC on gelatin as a feeder. (**a**) MCF7 cell models; (**b**) MDA-MB-231 cell models; (**c**) SK-BR-3 cell models. Designed 3D cell models: 3De—homotypic model consisting of BC cells; 3D-2 end—heterotypic model consisting of BC and endothelial cells; 3D-3 BrC4f—heterotypic model consisting of BC, endothelial cells and BrC4f CAFs; 3D-3 BN 120f—heterotypic model consisting of BC, endothelial cells and BN120f normal fibroblasts. Blue arrows indicate the distance of cell exit. The scale bar is 100 µm.

Fibronectin, a major protein of the ECM, is critical for cell migration by mediating cell adhesion through interactions with integrins, which then trigger signaling pathways that organize the cytoskeleton [33]. Vimentin, a cytoplasmic intermediate filament, has been demonstrated to support adhesion-dependent migration, enhance cell polarity, modulate cell cohesion, and maintain cell–cell contacts during collective migration [34]. In order to assess alterations in the TME cell-added BC spheroid model, a Western blot (WB) analysis of cell lysates was performed. Large glycoproteins such as fibronectin, which vary in size due to alternative splicing, are visible on WB as separate bands, typically around 220–270 kDa, reflecting different combinations of mRNAs (EDA, EDB, IIICS) [35]. Our analysis by WB showing fibronectin protein bands resolved at 272 kDa and 250 kDa (https://www.abcam.com/en-us/technical-resources/target-tips/fibronectinfn1, accessed on 22 November 2025) in SDS-PAGE (Figure 4b). The 250 kDa subunit has been identified as the most prevalent size monomer of fibronectin [36]. It is the primary constituent of both soluble plasma fibronectin and insoluble cellular fibronectin [37,38]. This larger form as 272 kDa is one of numerous isoforms produced by the process of alternative splicing of a single gene. The discrepancy in size is typically attributable to the incorporation of specific additional domains (EDA and EDB). As demonstrated in Figure 3b, the highest level of large fibronectin was observed in the sample obtained from CAFs spheroids (at twice the level of the sample from normal fibroblasts). The level of fibronectin protein, such as 272 kDa and 250 kDa, in homotypic and heterotypic spheroids (tumor-endothelial cells) from MCF7 cells was found to be low, and low levels were also identified in spheroids from MDA-MB-231 and SK-BR-3. As illustrated in Figure S4a, the analysis of tumor cells revealed low fibronectin expression in 2D, with the MDA-MB-231 and SK-BR-3 cell lines demonstrating lack of expression. However, when monocultured in 3D, it can be seen that two forms of fibronectin protein are synthesized, with the highest levels observed among tumor cells in MDA-MB-231 and SK-BR-3. This finding is consistent with the existing literature and is specifically related to the culture conditions [39]. Conversely, in complete heterotypic spheroids (3D-3), the fibronectin level exhibited a marked increase in comparison with both homotypic spheroids and heterotypic spheroids (tumor–endothelial cells) across all tumor cell types. This increase was most pronounced for MDA-MB-231 and SK-BR-3 (Figure 3b). The predominant contribution to this process is most likely derived from fibroblast populations (Figure S4). It is noteworthy that the 250 kDa fibronectin isoform manifested exclusively in heterotypic spheroid models, suggesting that its expression may be attributable to heterotypic interactions rather than merely the presence of fibroblasts. Levels of vimentin expression were found to be lowest in homotypic tumor spheroids and heterotypic spheroids (tumor-endothelial cells) derived from MCF7 and SK-BR-3 cells, and highest in MDA-MB-231 cells. Observations were made regarding elevated vimentin levels in all types of spheroids derived from MDA-MB-231 tumor cells. For the 3D-3 model from MCF7 and SK-BR-3 cells, co-culture with stromal and endothelial cells increased vimentin levels in the model (Figure 3b). The alterations in the ECM protein levels in the cell spheroid models were concomitant with the results of the functional assay for cell re-adhesion and migration (Figure 3a). It can be concluded that the observed phenotype cannot be explained solely by the “intrinsic profile” of fibroblasts. In order to understand the process under investigation, the following two factors must be considered: (a) The profile of the tumor cells is subject to radical change. It has been observed that the emergence of a qualitatively novel isoform is contingent upon intercellular cross-flow. The combination of three-dimensional architecture and heterotypic signals functions as an inducer, with fibroblasts playing a key role in this process.

The contribution of matrix stiffness to migration and invasion can manifest itself in different ways, including stimulation of tumor and stromal cells, depending on the environment. Adhesive plastic for cell culture is generally considered a rigid material under standard culture conditions [7]. Thus, on a stiff matrix such as culture plastic for aggressive cell lines of BC (SK-BR-3, MDA-MB-231), the tumor stroma (fibroblasts, endothelium) acts as an “accomplice” in enhancing aggregation and the formation of dense spheroids, followed by activation of invasive potential due to increased ECM protein production by stromal cells, regardless of their type. Conversely, for less aggressive cell lines of BC (MCF7), the stroma can act as a “restraining factor”, creating a barrier that limits the motility and proliferation of MCF7 cells, which do not initially exhibit high invasive potential. Subsequently, mechanobiological studies were conducted to investigate the influence of the biophysical properties of the substrate upon the addition of fibroblasts and endothelial cells on the invasiveness and motility of tumor cells. This investigation used feeders of varying degrees of stiffness and elasticity (gelatine and Matrigel^TM^).

### 3.3. Migration/Invasion of Cancer Cells Regulated by Matrix Stiffness

**Figure 5:**
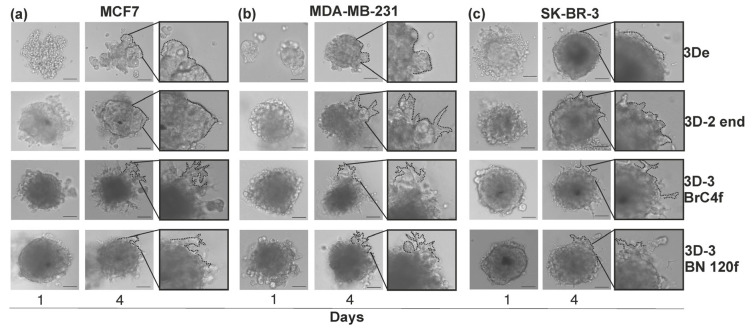
Invasion cells from 3D cellular models of BC using Matrigel^TM^ as a feeder. (**a**) MCF7 cell models; (**b**) MDA-MB-231 cell models; (**c**) SK-BR-3 cell models. Designed 3D cell models: 3De—homotypic model consisting of BC cells; 3D-2 end—heterotypic model consisting of BC and endothelial cells; 3D-3 BrC4f—heterotypic model consisting of BC, endothelial cells and BrC4f CAFs; 3D-3 BN120f—heterotypic model consisting of BC, endothelial cells and BN 120f normal fibroblasts. Invasive growths derived from spheroid cells are denoted by a dotted line. The scale bar is 100 µm.

Two-dimensional (2D) cell migration without physical barriers on a 2D matrix and 3D cell invasion with matrix degradation or deformation in a 3D matrix are regulated differently depending on matrix stiffness [7]. Within the domain of experimental cell biology, the terms “migration” and “invasion” are distinctly defined and serve different purposes [26]. The definition of migration is the directed movement of cells on a substrate, which can be a natural hydrogel. In order to assess cell migration abilities in vitro, the use of 2% gelatin as a substrate is recommended, as it is a porous, inert material in which tumor cells are able to spread unhindered. This substrate was therefore selected for the present study (Figure 4). Invasion can be defined as the process by which a cell moves through a 3D matrix. This is accompanied by a rearrangement of the 3D environment for cell exit. A seminal technique that has gained wide popularity in the study of cell invasion in vitro is the test in substrate from Matrigel^TM^, a mixture of gel-like materials containing ECM proteins (Figure 5). Cells that possess invasive potential have been observed to disrupt Matrigel™, forming pores within its structure. Through the utilization of these openings, the cells have demonstrated an ability to penetrate the substrate profoundly from the spheroids, resulting in the formation of star-shaped protrusions [40]. In the present study, the analysis of cell invasion was undertaken utilizing a substrate comprising diluted Matrigel^TM^, mixed with culture medium at a 1:1 ratio.

As demonstrated in Figure 5, the process of cell exit from the spheroid on a gelatin substrate is contingent upon the complexity of the 3D cell model. Homotypic spheroid models are distinguished by uniform exit without the formation of migration vectors, in contrast to heterotypic spheroid models. The greatest increase in cell motility was observed in heterotypic spheroid models consisting of tumor, endothelial cells and fibroblasts. Concurrently, the “motility” of cell from the 3D-3 model with CAFs is characterized by the longest exit distance (blue arrows). By the eighth day of the experiment, the homotypic spheroid had practically disintegrated, and the cells continued to migrate, which also indirectly confirmed the viability of the cells in the spheroid. Furthermore, cells persisted in migrating from heterotypic spheroid models, with the presence of discernible lumps, which are likely to represent an internal necrotic core and non-cellular components, most likely comprising extracellular matrix proteins (Figure S5).

It was observed that co-culturing endothelial cells with HER2+ SK-BR-3 or MDA-MB-231 in 3D-2 resulted in an enhancement of the invasive potential of cells from 3D-2 spheroids (Figure 5b,c). The study revealed that co-culturing all types of breast cancer cells with fibroblasts enhanced the invasive properties of tumor cells, demonstrating a moderate preferential effect of CAFs BrC4f compared to normal BN120f in the 3D-3 models. In parallel with these findings, evidence was presented demonstrating the use of Matrigel^TM^ as a nutrient agent, thereby enhancing the process of spheroid compaction. As of day 4, the trend of invasive potential remained consistent (Figure S5).

Stromal cells in heterotypic spheroid model, including CAFs, have been shown to activate tumor cells, promote the transition of cancer cells into a dormant state and reactivation, and stimulate the invasion, survival and proliferation of tumor cells, ultimately supporting metastatic growth.

### 3.4. Protein–Protein Interaction Network of Cytokines and Growth Factors via Search Tool for the Retrieval of Interacting Genes/Proteins of Database

In an attempt to enhance comprehension of the intricacies of cytokine signaling within the context of the breast cancer microenvironment, an analysis of protein–protein interaction was conducted utilizing the STRING (Search Tool for the Retrieval of Interacting Genes/Proteins) database [41]. This database integrates information pertaining to predicted and experimentally validated protein interactions, drawing upon the cytokine/chemokine inventory of the human cytokine multiplex screening panel (48-plex, Bio-Rad). The molecules under consideration have been grouped according to their functional relationships. This has allowed the identification of key nodes that control critical processes such as angiogenesis, invasion, stem cell maintenance, and the formation of an immunosuppressive environment.

**Figure 6:**
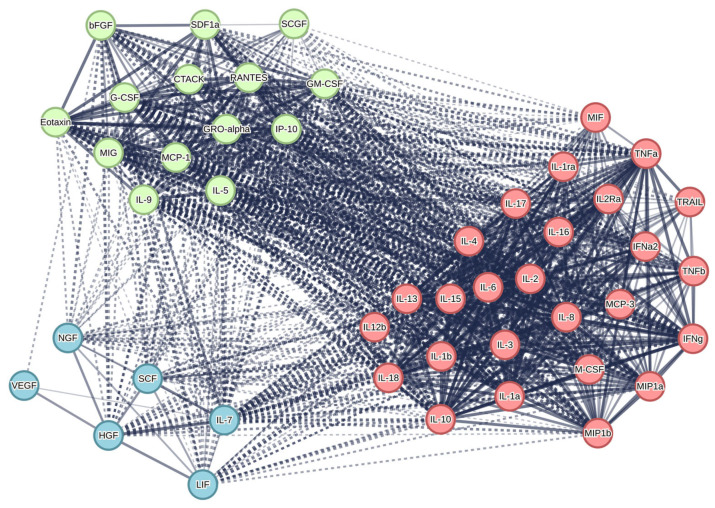
Protein–Protein interaction network of cytokines and growth factors. The STRING interaction network displays cytokines and growth factors organized into three functional clusters (k-means algorithm) based on their biological roles and interaction patterns. The first cluster (red nodes) pertains to the interaction between cytokines and their receptors. The second cluster (green nodes) concerns the binding of chemokine receptors to chemokines. The third cluster (blue nodes) is associated with IL-7 and growth factors that are linked to cell development and survival.

Network analysis of 48 cytokines and growth factors revealed a high degree of integration and functionality among the molecules under study. The network consists of 906 interaction edges with an average node degree of 39.4, indicating a dense interconnection between proteins. It is critically important that the observed number of interactions significantly exceeds the expected number (906 versus 118), as evidenced by the statistical significance of *p* < 1.0 × 10^−16^, indicating that the probability of such a number of interactions occurring randomly in the genome is virtually impossible. The high local clustering coefficient (0.935) demonstrates that proteins in the network are organized into dense functional modules, where neighboring nodes often interact with each other. The combination of these indicators—a high degree of nodes, significant enrichment of protein–protein interaction (PPI), and pronounced clustering—convincingly demonstrates that the studied set of cytokines and chemokines is not a random collection, but represents a biologically integrated system of molecules that actively interact in multilevel signaling networks regulating key processes in the tumor microenvironment. The application of the k-means algorithm to the clustering of the identified protein network enabled the identification of three functionally distinct groups of cytokines and growth factors (Figure 6).

Cluster 1 (red): Innate immune response cytokines. The first functional cluster comprises classical cytokines and chemokines of innate immunity, including interferons (IFN-α, IFN-γ), interleukins (IL-1α, IL-1β, IL-6, IL-8, IL-10), TNF-α and chemokines (MCP-1, MCP-3, RANTES, MIP-1α, MIP-1β). These molecules have been demonstrated to coordinate the inflammatory response, activate macrophages, attract immune cells, and regulate innate immunity. Within the context of breast cancer, these cytokines frequently promote a pro-inflammatory and immunosuppressive microenvironment, thereby supporting tumor growth [24].

Cluster 2 (green): Chemokines, growth factors and recruitment molecules. The second cluster encompasses chemokines and growth factors implicated in the recruitment of microenvironmental cells and the regulation of angiogenesis. This cluster includes SDF-1α, colony-stimulating factors (G-CSF, GM-CSF), IL-5, IL-9, IP-10, MIG, RANTES, SCGF-β and basic fibroblast growth factor (bFGF). It has been established that SDF-1α, through its receptor CXCR4, activates critical signaling pathways (PI3K/Akt, MAPK/ERK, JAK/STAT3) that promote tumor growth, invasion and migration. SCGF-β has been identified as a critical factor in the regulation of the tumor stem cell population. The synergy between SDF-1 and other factors within this cluster engenders a positive feedback loop that fosters angiogenesis, tumor progression and metastasis [42,43].

Cluster 3 (blue): IL-7 and related cell development factors. This cluster combines IL-7 with the growth factors HGF, LIF, NGF and SCF (stem cell factor) and includes VEGF. This clustering can be explained by the functional convergence of these molecules in regulating cell development, survival, and differentiation. IL-7 was initially characterized as a critical cytokine for B and T lymphocyte development, regulating the proliferation, survival and differentiation of lymphoid progenitors by activating the JAK-STAT5 pathway. However, recent studies have shown that fibroblasts expressing IL-7 are localized at tumor margins and interact directly with cancer cells, promoting their stemness and aggressiveness. Therefore, IL-7 acts as both an immune modulator and an important component of the microenvironment that supports the cancer cell phenotype [44].

### 3.5. The Analysis of the Secretory Profile of a Heterotypic Spheroid Model

**Figure 7:**
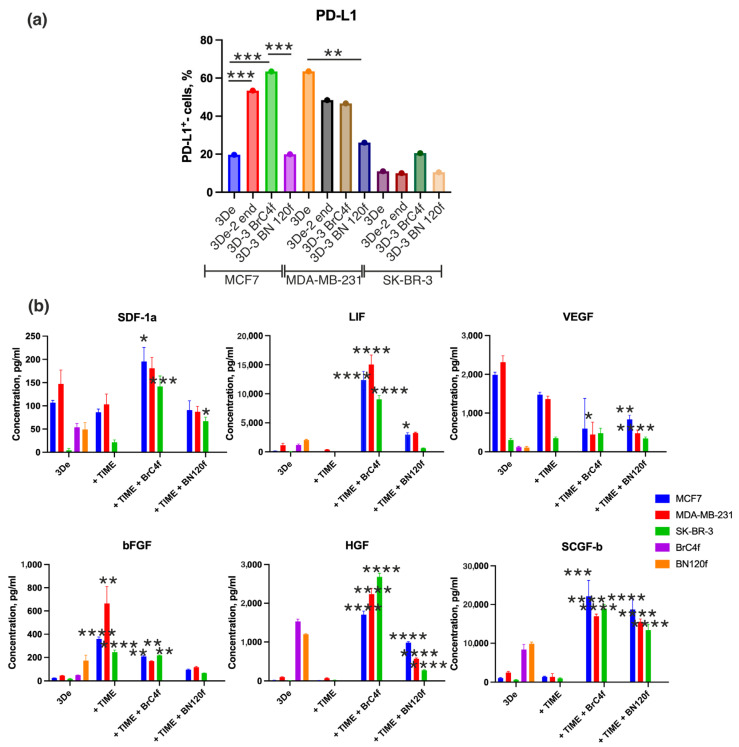
Characterization of the immunosuppressive TME in spheroid models of BC. (**a**) The relative contribution of the PD-L1+ subpopulations in spheroid models of BC. Bar graph showing the percentage of PD-L1+ cells detected by flow cytometry. (**b**) The estimation of cytokines SDF-1a, LIF, VEGF, bFGF, HGF, SCGH-b by 48-plex human cytokine array in conditioned medium. Data represented as mean ± SEM from three wells of conditioned medium. The difference between the experimental groups compare with control (homotypic spheroid) was statistically significant at * *p* < 0.05; ** *p* < 0.01; *** *p* < 0.001; **** *p* < 0.0001 (non-parametric Mann–Whitney U-test).

PD-L1 (programmed death ligand 1) is a protein that is utilized by cancer cells to evade the immune system, while cytokines are signaling molecules that can influence PD-L1 expression and tumor immunity [44,45]. The frequency of PD-L1+ cells exhibited variability within homotopic breast cancer spheroids, with the highest number of positive cells observed in the MDA-MB-231 (Figure 7a). The addition of endothelial cells and CAFs, but not normal fibroblasts, increased the frequency of PD-L1+ cells, which contributes to subsequent tumor evasion from immune control. Moreover, comparative analysis of the PD-L1-positive population in 2D and 3D cell models demonstrated that the level of positive cells did not correlate between 2D and 3D cultures and that this population increased in heterotypic spheroids, whereas in monolayer fibroblasts it was only weakly positive. The highest level was observed in the MDA-MB-231 cell line, but in a heterotypic 3D culture from this tumor line, this level decreased (Figure S4). To study the plasticity of luminal, HER2+ and triple-negative BC cell subpopulations with different degrees of CD44 and CD24 expression (Figure S6), flow cytometry was used. Concurrently, despite a parallel tendency in CD24 and CD44 content between 2D and 3D models, it is evident that the proportion of such cells escalates in 3D cultivation. These alterations are not contingent on the content of CD24 and CD44 in stromal cell cultures (Figure S4). Within the homo- and heterotypic MDA-MB-231 model, a minor, low-differentiated subpopulation of CD24^−^CD44^+^ cancer stem cells was found to be enriched compared to the luminal subtype MCF7 and the HER2+ SK-BR3 subtype. This correlates with known evidence of the greater aggressiveness, metastasis and drug resistance of triple-negative BC due to increased cancer stem cells (CSCs) content [46]. CSCs usually constitute a small fraction of tumor cells, but in the case of SK-BR3, we observed an increase in this minor CSCs fraction in the presence of fibroblasts. At the same time, changes in other subsets were observed: a significant decrease in the more differentiated CD24^+^CD44^−^ subset, and an increase in the CD24^−^CD44^−^ subset. This generally reflects the high plasticity of SK-BR-3 cells. MCF7 luminal-type models, however, are much less susceptible to changes in subsets in the presence of fibroblast signals. This is consistent with existing data on the predominance of estrogen receptor (ER)-associated mechanisms in maintaining the luminal phenotype and blocking epithelial–mesenchymal transition (EMT) with a lack of CSCs enrichment [47]. We also observed higher levels of CD44 expression in MCF7 and MDA-MB-231 cells than in SK-BR-3 cells. This difference in expression levels correlates with higher PD-L1 expression levels in MCF7 and MDA-MB-231 compared to SK-BR-3 (Figure 7a). Our data are consistent with the established mechanism whereby PD-L1 expression is initiated through CD44 binding to PD-L1 regulatory regions. This results in a correlation between PD-L1 and CD44 expression in BC samples [48].

Cytokines produced by various cellular components of the TME and play a key role in the regulation of progression, and metastasis [49]. In order to characterize the functional state of cells in the models, an analysis of the secretory profile of cultures was conducted, with particular attention paid to cytokines and chemokines associated with the formation of an immunosuppressive environment and an invasive phenotype (Figure 3b, Figure 4 and Figure S6). In the subsequent phase of the study, the alterations in the levels of the cytokines/chemokines as SDF-1, LIF, VEGF, bFGF, HGF, and SCGF-β from clusters 2 and 3 were the primary focus. Cluster 1 was excluded from the analysis (Figure 6). The rationale for this exclusion was that these factors formed a single signaling axis that integrated the processes of angiogenesis, stem cell survival, immunoregulation, and microenvironment remodelling in BC (Figure 7). A key discovery was a substantial increase in the secretion of LIF (leukemia inhibitory factor) and HGF (hepatocyte growth factor) in heterotypic spheroids with CAFs (BrC4f), irrespective of the molecular type of tumor cells. This finding is significant in the context of the regulation of PD-L1 expression in breast cancer, which is known to be controlled by oncogenic signaling pathways (JAK/STAT, MAPK, PI3K/Akt) and proinflammatory cytokines [50]. Concurrently, we observed remodeling of the angiogenic profile. Levels of VEGF (vascular endothelial growth factor) were found to be elevated in homotypic spheroids MCF7 and MDA-MB-231, with a decline observed as complexity of the models increased. Furthermore, heterotypic spheroids with BrC4f demonstrated an augmentation in SDF-1 (stromal cell-derived factor-1) secretion. The observed shift towards SDF-1 dominance may be indicative of a transition from classical angiogenesis to the activation of invasion and metastasis mechanisms through HIF1 activation, an increase in the cancer stem cell population, and the formation of an immunosuppressive microenvironment. It is also noteworthy that all heterotypic 3D models, irrespective of the stromal cell type, exhibited a characteristic increase in the level of SCGF-β (Stem Cell Growth Factor-β), which is consistent with the activation of cancer stem cell-associated programmers. This phenomenon is further compounded by bFGF, a potent inducer of angiogenesis that can directly stimulate endothelial cell proliferation (Figure 7b).

**Figure 8:**
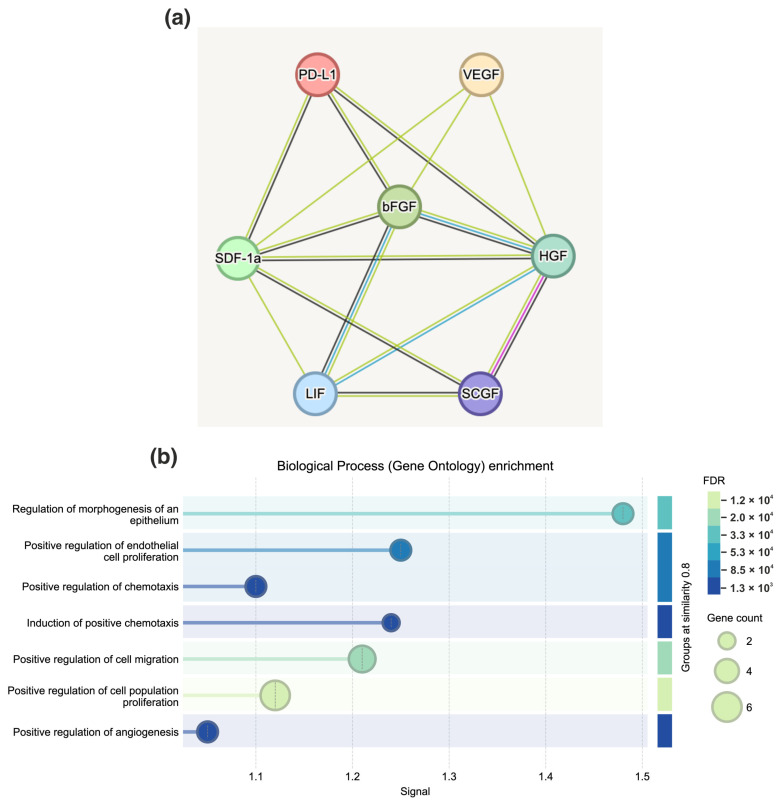
Analysis of the STRING protein interaction network and its functional interpretation cytokines SDF-1, LIF, VEGF, bFGF, HGF, and SCGF-β. (**a**) Focus on the relationship of VEGF with SDF-1a, bFGF, and HGF only when these are mentioned together in PubMed abstracts. Note that LIF is mentioned in PubMed abstracts in conjunction with SDF-1a only. VEGF and LIF are associated with PD-L1 only through SDF-1a, bFGF, and HGF, but never directly. (**b**) The Biological Process Gene Ontology enrichment diagram confirms the functional architecture of the network.

These models possess the capacity to reproduce a variety of phenotypes, and the format of the model itself (i.e., its ability to fit into a test tube) provides a wide range of opportunities for researching the characteristics of cancer development, metastasis, treatment, and resistance to therapy The hypothesis is proposed that the cytokines LIF, SDF-1, HGF, and SCGF-β may be associated with a regulatory role in PD-L1 expression by tumor cells, thereby opening up potential avenues for investigating mechanisms of immune evasion. This phenotype is characterized by a transition from VEGF-dependent angiogenesis to a strategy aimed at recruiting microenvironmental cells and activating the JAK/STAT3 and PI3K/Akt signaling pathways. This process ultimately stabilizes the PD-L1 protein on the membrane of tumor cells, rendering them “invisible” to the immune system (Figure 8).

## 4. Discussion

The tumor microenvironment (TME) is a complex system of heterogeneous stromal cells, including vascular cells (e.g., endothelial cells, smooth muscle cells, pericytes) that interact with each other to support tumor initiation, progression, drug resistance and metastasis [1,51]. Of these stromal components, cancer-associated fibroblasts (CAFs) have emerged as the predominant components within the TME, actively shaping multiple aspects of tumorigenesis, including cancer cell proliferation, invasiveness, and immune evasion. Specifically, CAFs have also been demonstrated to regulate the production of proangiogenic factors, thereby fueling neovascularization to support the metabolic demands of proliferating tumor cells. Furthermore, CAFs have the capacity to exert a direct or indirect influence on endothelial cell behavior and vascular architecture, which may, in turn, impact cancer progression [52].

The concept of “hallmarks of cancer” has evolved into a paradigm that guides all of modern oncology [53]. Hallmarks of Cancer can be viewed as another basis for the development of more complex, multicomponent and physiologically relevant cellular models, which, in turn, pave the way for the development of targeted and effective drug approaches. The evolution of cellular models is a direct response to a deeper understanding of cancer biology as a complex, multifactorial system, and research tools must match this complexity. Budhwani et al. provided a thorough review of contemporary cancer modelling platforms, encompassing in vitro, in vivo, ex vivo and in silico models. They traced the evolution of these models and emphasized the increasing demand for integrated ‘cancer supermodels’ that leverage their advantages and address their limitations [17].

Selecting the optimal method for forming spheroids is a fundamental step in the experimental planning process. Methods for producing individual spheroids offer the high level of reproducibility and standardization necessary for pharmacological and screening studies [54]. At the same time, mass production methods are an effective way of accumulating biomaterial for subsequent omics analyses and studies that tolerate greater dimensional heterogeneity. Therefore, experimental design must be based on a clear understanding of this trade-off, where the choice between productivity, relevance, and control over model parameters is dictated by research objectives [16]. The liquid layering on agarose, gelatin or Matrigel^TM^ methods did not enable the formation of distinct, compact heterotypic spheroids in the majority of the tested breast cancer cell lines [30]. U-bottom plates with ultra-low adhesion promote cell proximity and adhesion, resulting in more compact structures. These plates are also ideal for screening experiments, as each well can be used to test different conditions or compounds independently. In the present study, the previously developed heterotypic spheroid model of BC was complexified by the incorporation of endothelial cells as a component of tumor vascularization [22]. Utilizing open spatial transcriptomics data, we evaluated the ratios of tumor, stromal, and endothelial cells in clinical samples to ascertain the cell concentration in the model [55,56,57]. Also, the cell proliferation rate was considered when constructing the cell spheroid model (Figure 1a). Piwocka et al. also incorporated spatial transcriptomics data into their study, yet they did not consider the initial proliferation rate of microenvironment cells, which impacts the quantitative ratio of cells in the research 3D model and the predominance of the tumor epithelial component [4]. In a previous study, the addition of stromal cells at 1:1 and 1:4 (tumor:stromal) ratios was evaluated, and it was found that the addition of stromal cells at the 1:1 ratio was insufficient due to the lower growth rate of stromal cells [21,22]. In the case of endothelial cells, the cell concentration was increased based on the study cell ratio [4,58] and baseline cell proliferation rate. Our previous study demonstrated that co-culture of tumor and stromal cells was associated with a higher propensity to form rounded and structured spheroids compared to those formed of homotypic spheroid [21,22]. The present study found that this tendency was sustained, with stromal cells, as opposed to endothelial cells, being the primary contributors to the development of the heterotypic BC configuration (Figure 1b, Figures S1 and S2a). Fibroblasts are required to maintain the endothelial cell population by secreting ECM proteins [59] and ensure cell viability in the heterotypic spheroid model without the addition of VEGF (Figure 1c and Figure S2), consistent with the results of [58]. Confocal microscopy analysis revealed that EC organization in the heterotypic spheroid model is formed into vessel-like structures (Figure 2). The findings of this study demonstrated a correlation between the type of tumor cells utilized in the SK-BR-3 ≥ MCF-7 > MDA-MB-231 series and a decline in both tumor size and spheroid heterogeneity [21].

A critical step in the development of relevant 3D cell models is their ability to reproduce key histoarchitectonic features of native tumor tissue [60]. The developed heterotypic spheroids reproduce a key histological feature of invasive carcinoma-tumor-stromal separation. Dense tumor nests are distinctly demarcated from the stromal compartment, where endothelial cells form primitive pseudovascular networks exclusively within fibroblast strands, reflecting the architecture of native tumor tissue. The formed structures demonstrate the morphology of pathological angiogenesis, as evidenced by chaotic branching, a lack of hierarchy, and unstable intercellular contacts. Furthermore, the differential effect of the modelled microenvironment—wherein stromal and endothelial cells significantly enhanced the adhesive and migratory potential of the aggressive MDA-MB-231 and SK-BR-3 cells, whilst having no effect on the less aggressive MCF-7 cells—directly reflects the clinical behavior of these molecular subtypes. The correspondence between the results obtained in the model and those known to occur in vivo biology serves to emphasize the physiological relevance of the system for the study of specific, subtype-dependent interactions between tumor and stroma. In spheroids containing fibroblasts and HUVECs [61], endothelial cells proliferation was closely associated with fibroblasts, and HUVEC island formation was observed in the spheroid core in close association with fibroblasts [62,63]. Ascheid et al. demonstrated that pseudovessels were present within fibroblast bands, thereby avoiding contact with tumor cells [64]. This is a histological feature of clear separation of tumor cells and stroma, which is found in many carcinomas. It is well demonstrated that tumors require the recruitment of new blood vessels to meet their ever-increasing metabolic demands [31] and that tumor angiogenesis is essential for metastasis [65]. It has been observed that cells cultivated in spheroids exhibit a metabolic response analogous to that of epithelial cells in vivo [15]. The reattachment test [66,67,68] was utilized to evaluate the adhesion capabilities of cells. It was demonstrated that endothelial and stromal cells exhibited distinct behaviors for three molecular types of BC cells (Figure 3). This investigation demonstrated that, in contrast to cellular models from triple-negative BC MDA-MB-231 (TNBC) and HER2-positive SKBR3 (HER2+) cells, the incorporation of endothelial or stromal cells in spheroids from hormone-positive MCF7 (ER+) cell did not exert any influence on cell adhesion and proliferation.

Fibronectin is a glycoprotein whose size ranges from 230 to 270 kDa and usually exists as a dimer, covalently linked by a pair of disulfide bonds at the C-termini. The 250kDa band corresponds to the canonical monomer, which is devoid of the EDA and EDB domains. This isoform is constituted by the soluble plasma fibronectin produced by hepatocytes and is also a prevalent form of cellular fibronectin [35,36]. It is noteworthy that in the models under consideration, a 250 kDa signal was detected exclusively in heterotypic spheroids containing fibroblasts (CAFs or normal fibroblasts). This finding indicates that the expression of this protein is not solely derived from fibroblast cells, but rather is induced or significantly increased by heterotypic interactions with tumor cells. The 272 kDa band is consistent with a monomer containing both the EDA and EDB extra domains, a hallmark of cellular fibronectin produced during active tissue remodeling, fibrosis, and cancer progression [69,70]. The most intense signal for this isoform was observed in spheroids incorporating CAFs, aligning with their established pro-fibrotic and tumor-promoting phenotype. Heterotypic spheroids comprising normal fibroblasts exhibited elevated levels in comparison to tumor-only models, though to a lesser extent than CAF-containing spheroids (Figure 3).

Activated stromal cells in various organs prepare the “ground” for metastatic spread [71]. A temporal analysis of stromal cell functions reveals that they promote the chemoattraction of disseminated cancer cells to the site of metastasis, maintain the quiescent state of cancer cells, and mediate the exit from this state [72]. Furthermore, stromal cells have been shown to promote the invasion, survival, and proliferation of tumor cells, in addition to modulating the formation of a suppressive immune microenvironment at sites of metastasis. This, in turn, supports metastatic growth. Fibroblast activation and increased ECM stiffness create a feedback loop within the tumor microenvironment that promotes cancer cell invasion and migration [73]. The results of the present study demonstrate that heterotypic spheroid models are characterized by the activation of invalid and migratory features on gelatin and matrix gel (Figure 4 and Figure 5).

Breast cancer is a highly heterogeneous disease, presenting diverse histological and biological properties due to genetic, epigenetic, and transcriptomic alterations that impact diagnosis, treatment, and prognosis [74]. Luminal A (ER+) is associated with a favourable prognosis, primarily due to its tendency to exhibit slower growth rates compared to other subtypes of BC and a high response rate to hormonal therapy [75]. In ER+ tumor, in contrast to the assumption that is commonly made for normal breast tissue, where cross-talk with the adjacent stroma is important, estrogens have been shown to directly influence the proliferation of ER+ tumor cells [76,77]. Furthermore, tumor progression has been observed to be associated with stromal changes that promote the malignant phenotype by secreting additional growth-promoting, angiogenic, immunoregulatory, and proinvasive soluble factors [78]. However, the metastatic potential of such estrogen-dependent cells is low (Figure 3a and Figure 4), and tumor progression is more likely to be associated with the acquisition of resistance to therapy [79]. You et al. demonstrated that fibronectin expression is increased by PI-3K/Akt activation in tamoxifen-resistant breast cancer cells [80]. The heterotypic spheroid model from MCF7 cells is characterized by an augmentation in fibronectin synthesis, irrespective of the stromal cell type (Figure 3b). This increase is hypothesized to be a predictor of the development of resistance to therapy in the model and forms the focus of future research. Recent studies have demonstrated that PDGF receptors and their corresponding ligands play a pivotal role in the modulation of tumor cell lineage, determining whether a tumor exhibits a luminal or basal characteristics. This, in turn, determines its response to endocrine therapy [81]. As demonstrated by Roswall et al., the presence of CAFs has been identified as a determining factor in the classification of BC molecular subtypes [82]. Indeed, the results demonstrate that heterotypic spheroid models from MCF7 cells are characterized by a decrease in PDGF secretion levels (Figure S6). Furthermore, the development of resistance to therapy can occur due to an increase in the proportion of CSCs [83]. The cytokine LIF is a key regulator of CSCs; it plays a role in stem cell maintenance, regulates self-renewal and pluripotency, and is also associated with chemoresistance (Figure S6) [84]. As demonstrated in Figure 7, the level of LIF secretion is increased fold in the 3D model with CAFs. Martinez-Outschoorn et al. demonstrated that CAFs induce resistance to tamoxifen by increasing mitochondrial activity in breast cancer cells [85]. Huang showed that paracrine stromal signaling results in ER downregulation in MCF-7 and T47D cells [86]. Furthermore, estrogen can increase SDF1 (also known as CXCL12) production in cancer cells and the TME, while the SDF1/CXCR4 signaling pathway has been shown to activate ER and promote hormone-independent tumor growth [87]. Therefore, despite the fact that MCF7 cells do not acquire aggressive invasive potential when co-cultured with microenvironment cells, the interaction of CAFs and endothelial cells mediated by paracrine signals (LIF, SDF-1, HGF, SCGF-β) can be considered a potential therapeutic target for indirectly affecting the tumor epithelial compartment (Figure 5). In ER+ tumors, estrogen has been shown to stimulate the secretion of MCP1 (also known as CCL2), thereby promoting the recruitment of certain immune populations, such as myeloid-derived suppressor cells, and enhancing their innate immunosuppressive activity [88], a finding that is also consistent with the results presented in this study (Figure S6). As demonstrated previously, the presence of NK cell killer activity in a heterotypic spheroid model of MCF7 and CAF is reduced [22].

Two other molecular types of BC (TNBC and HER2+) are aggressive forms of cancer and are characterized by more rapid tumor progression and metastasis [89]. In contrast to the cells of the hormone-dependent MCF7 line, the endothelial and stromal cells of the MDA-MB-231 and SK-BR-3 cells exert a direct effect on the tumor cells, regulating their adhesion and migration potential (Figure 3a, Figure 4 and Figure 5). It has been established that CAFs are responsible for the production of a substantial quantity of ECM [90], which in turn can result in the formation of a dense fibrous capsule surrounding the spheroid. In the course of conducting a “reattachment test”, a “physical barrier” is observed to manifest itself, which mechanically hinders the exit of cells from the spheroid. This is observed as a “delayed phase of adhesion”. However, in the long term, the same niche created by CAFs provides the tumor with signals (HGF, bFGF) and promotes invasion and resistance. Fibronectin has been identified as an important component of metastatic niches in BC [91] and was found to be highly expressed in primary and metastatic TNBC and HER2+ tumors [81]. Heterotypic spheroid models from MDA-MB-231 and SK-BR-3 cells are characterized by increased synthesis of fibronectin and vimentin, which is also accompanied by increased aggregation and formation of dense spheroids with subsequent activation of invasive potential due to an increase in ECM proteins by stromal cells, regardless of their type (Figure 3). Tunalı et al. demonstrated that the positive feedback loop driven by fibronectin and IL-1β maintains the inflammatory microenvironment in breast cancer [92], a finding that is also consistent with the results presented in this study (Figure S5). Zarychta et al. showed that SDF-1α in invasive breast cancer has been shown to be associated with vasculoangiogenic factors and to possess prognostic significance [43]. VEGF has been demonstrated to promote the development of new blood vessels and carcinogenesis through the SDF-1α signaling pathway [93]. In homotypic spheroids from MDA-MB-231, high levels of VEGF were recorded, which decreased with increasing complexity of the models (Figure 7b). However, in heterotypic spheroids with CAFs BrC4f, increased SDF-1 secretion was observed. It has been established that the collaboration of SDF-1 and VEGF instigates a positive feedback loop that fosters angiogenesis, tumor progression and metastasis [94,95]. Moreover, in BC cell lines, the activation of the HGF pathway through binding of HGF to its receptor c-MET can result in increased cell survival, proliferation, and resistance to cancer inhibitors. In the context of breast tumors, clinical studies have identified a correlation between HGF pathway activation (as determined by c-MET overexpression) and increased tumor size, high tumor grade, and distant metastasis [96]. Although HGF-positive tumors were common among TNBC, they were a defining feature among basal-like tumors [97]. Furthermore, HGF c/MET expression has been demonstrated to impact prognosis, particularly in cases of HER2-overexpressing tumors, through resistance to HER2-targeted therapy [98]. As demonstrated in Figure 7b, a substantial augmentation in its secretion was observed in heterotypic spheroids with CAFs BrC4f, irrespective of the molecular type of tumor cells. On the one hand, HGF binds to its receptor, c-Met, thereby triggering signaling cascades including PI3K/Akt and MAPK/ERK [99]. In addition, the c-Met pathway has been shown to activate STAT3, thus creating a synergistic effect with LIF [100]. The present study demonstrates that activated STAT3 directly binds to the promoter of the CD274 gene (encoding PD-L1) and turns on its transcription [101,102]. On the other hand, SDF-1 activates the PI3K/Akt pathway via the CXCR4 receptor [103]. Akt has been demonstrated to not only stimulate PD-L1 production, but also to phosphorylate the PD-L1 protein itself, thereby preventing its degradation within the cell and increasing its stability on the surface [104]. In contrast, within the heterotypic spheroid model derived from MDA-MB-231, a decline in the proportion of PD-L1-positive cells was observed upon co-culturing with fibroblasts (Figure S4). One of the main TNBC cell models used in the article is MDA-MB-231, known for its overexpression of PD-L1. However, only 15–30% of primary TNBC tumors express PD-L1, which calls into question the relevance of the obtained data for practical conclusions [105]. It is noteworthy that CD44 has been identified as a potential target for PD-L1 suppression of BC function. CD44 has been demonstrated to activate transcription of the PD-L1 gene by an intracytoplasmic domain (ICD) binding to the consensus CD44-ICD-binding site of the PD-L1 regulatory region [48]. Furthermore, CD44-ICD has been shown to recognize the promoter region of the PD-L1 gene. CD44 activates transcription of the PD-L1 gene, thus indicating a positive correlation between CD44 and PD-L1 expression at the level of mRNA and proteins in BC. Changes in PD-L1 expression were detected through the observation of an increase in PD-L1+ tumor cells in the presence of endothelial cells and CAFs. This finding aligns with established data on the role of stromal cells in hindering immune responses. Consequently, our state-of-the-art 3D models can be utilized to assess the biological activity of novel multi-target molecules designed to interact with CD44 and PD-L1, with the objective of eradicating aggressive CD44^+^ populations and augmenting the efficacy of immune checkpoint inhibitors. There is a possibility that the phenomenon under discussion may be primarily associated with the TME and stromal cells. It has been demonstrated that the heterogeneity of CAF subpopulations within the TME can also differentially regulate the immunosuppressive environment of the tumor [106]. The BrC4f cell line was previously characterized as a subpopulation of matrix-producing fibroblasts that do not exhibit the immunosuppressive phenotype [26]. The synthesis of fibronectin and vimentin in this 3D model is increased, ensuring the formation of a dense physical barrier and a subsequent metastatic niche (Figure 3, Figure 4 and Figure 5).

Utilizing STRING protein–protein interaction network analysis and their functional interpretation, the focus was directed towards cytokines SDF-1, LIF, VEGF, bFGF, HGF, and SCGF-β that are less frequently analyzed in the immunosuppressive tumor microenvironment, which form a unified signaling axis integrating angiogenesis, stem cell survival, immunoregulation, and microenvironmental remodeling in BC (Figure 8). As demonstrated by the enrichment data from the Biological Process and Molecular Function gene ontology, the central role of SDF-1a as a coordination node is highlighted. SDF-1a has been shown to act as a hub, connecting two functionally distinct protein subgroups. According to the PubMed database, SDF-1a has been identified as a selective mediator of interactions between pro-angiogenic factors, thereby demonstrating direct connections with all six other network components. In the context of the joint scientific citation, VEGF, bFGF, and HGF form a synergistic triad that is mediated by SDF-1α [43,107,108,109]. The presented network demonstrates a hierarchically organized architecture of interactions. The initial level is defined as follows: VEGF, bFGF, and HGF have been identified as independent initiators of angiogenesis. Secondary level: The SDF-1a and its receptor CXCR4 have been shown to synchronize the action of primary factors through chemokine signaling. Tertiary peripheral level: LIF exerts an indirect effect on the network by stimulating the secretion of other members of the triad, while PD-L1 modulates the local immune environment to promote an angiogenic microenvironment. Consequently, the network can be conceptualized as a multi-level regulatory module, in which the positioning of each protein reflects its functional role in coordinating angiogenesis, morphogenesis and immunological processes in tissue structures. Further analysis of the activation of signaling pathways using heterotypic spheroid models has the potential to inform the development of more effective combination therapies.

## 5. Conclusions

In recent studies, the formation and growth of mono- and heterogeneous spheroids composed of BN120f fibroblasts and breast cancer cells, including ER+/PR+ MCF7, HER2+ SK-BR-3, and ER-/PR-/HER2-MDA-MB-231, have been thoroughly characterized [21,22]. In the aforementioned study, the effects of 17β-estradiol (E2) on spheroids were examined, and it was observed that E2 induced an increase in tumor cell proliferation. Concurrently, the expected MDA-MB-231 cells resistance to E2 was detected. However, simplified models have been employed in this previous study, in contrast to the more complex cell models developed in the current study. Our modern models utilize endothelial TIME-RFP cells and cancer-associated (BrC4f) or normal (BN120f) fibroblasts. In consequence of the evolution of novel cell models and the research conducted in this article, we have established sophisticated pertinent models for the testing of novel antitumor molecules, encompassing targeted drugs that are directed towards the CD44 cancer stem cell marker. This has exposed a discrepancy in the degree of differentiation of subpopulations in spheroids contingent upon their configuration (Figure S6).

We also managed to carry out a comprehensive characterization of the mobility and invasive properties of spheroids using extracellular matrices [7]. The presence of substrates revealed the potential of cell mobility in the presence of fibroblasts and endothelial cells, while the assessment of adhesion and proliferation by the xCELLigence RTCA system revealed mainly the effect of spheroid compaction in the presence of stromal cells. Thus, when conducting mechanobiological research, it is important to use substrates to ensure cell migration, which mainly depends on the rigidity of the matrix.

A tumor may be defined as a dynamic ecosystem that is heterogeneous and complex in its nature [81]. In the current understanding the TME of BC, the growth factors vascular endothelial growth factor (VEGF) and hepatocyte growth factor (HGF), as well as the regulators of cell survival and angiogenesis, insulin-like growth factor (IGF), play a key role in regulating progression and immunosuppression. These factors, in conjunction with IL-7, engage with PD-L1 via the JAK-STAT/PI3K-Akt cascades, thereby establishing a complex network of signaling pathways within the stroma and vascular component of the tumor. A growing volume of evidence suggests that a cytokine cluster comprising IL-7, HGF, LIF, and VEGF performs a pivotal function in orchestrating the activities of the TME through integrated signaling pathways. Research has demonstrated that IL-7-expressing fibroblasts are localized to the tumor border and physically interact with cancer cells, thereby promoting cancer stem cell and lymphoid development through the activation of JAK-STAT5 signaling [110]. HGF, secreted by CAFs, has been shown to stimulate VEGF expression, thereby enhancing angiogenesis, endothelial cell proliferation and invasive potential. This process involves EZH2 activation and the suppression of antiangiogenic factors [111]. The interplay between angiogenic signaling, including VEGF and HGF, and PD-L1 in tumor creates a synergistic axis where impaired vascular integrity and increased pericyte coverage correlate with increased PD-L1 levels, promoting immune escape [112]. The regulation of PD-L1 within the TME of BC is a complex process, involving multiple cytokine pathways, including IL-6-JAK-STAT3 and IFN-γ-JAK-STAT1 cascades. These pathways are activated by both cancer cells and inflammatory stromal components, emphasizing the necessity for a comprehensive understanding of the interplay between angiogenic (VEGF, HGF) and immunoregulatory (IL-7, LIF) signals to fully comprehend the mechanisms of immune escape [113]. Consequently, the IL-7/HGF/LIF/VEGF cluster has been identified as a functional module that orchestrates multiple processes, including development, angiogenesis, stem cell maintenance, and the establishment of an immunosuppressive phenotype within the breast cancer microenvironment. In the chemokine cluster, SDF-1, bFGF, and SCGF-β are key regulators that coordinate angiogenesis, the recruitment of endothelial progenitor cells, and the maintenance of CSCs in the TME. SDF-1α, secreted by CAFs via autocrine and paracrine mechanisms, activates CXCR4-dependent signaling. This, in turn, not only stimulates cancer cell invasiveness and migration through the activation of the PI3K/AKT pathway, but also promotes the formation of an immunosuppressive microenvironment by directly interacting with the PD-1/PD-L1 axes. This, in turn, attracts immune cells to tumorigenic areas where their effector function is suppressed through increased PD-L1 expression on the membranes of tumor and endothelial cells [114,115]. bFGF, through interaction with FGFR1-4, initiates proinflammatory cascades and antiapoptotic programmers in cancer cells [116,117]. SCGF-β, in its capacity as a regulator of the maintenance of hematopoietic and cancer stem cells, exerts a synergistic effect with SDF-1α and bFGF by establishing niches that promote the maintenance of cancer cell populations and the differentiation of myeloid cells into suppressor populations, thereby facilitating the development of myeloid-derived immunosuppression [118]. The remaining components of the cluster (CTACK, Eotaxin, G-CSF, GM-CSF, GRO-α, IL-5, IL-9, IP-10, MIG, RANTES) perform auxiliary functions in the recruitment and activation of immune cells and the increase in the chemokine gradient. However, they do not regulate the relationship between inflammation, angiogenesis and PD-L1-dependent immune escape.

**Figure 9:**
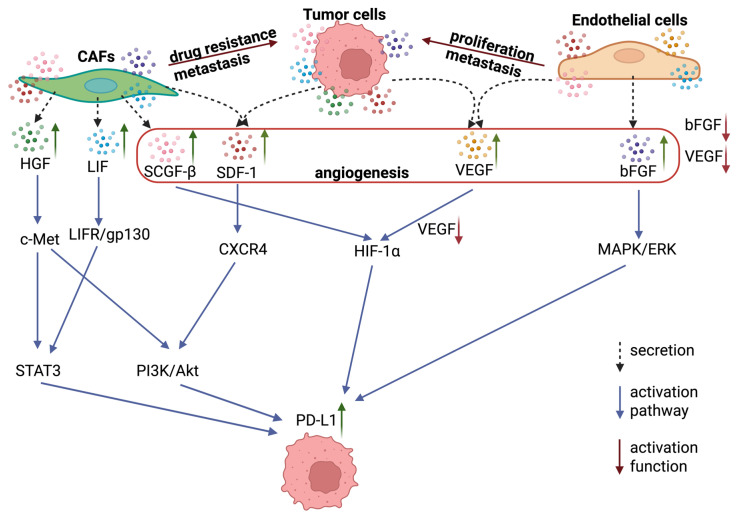
Possible mechanism of coordinated action of key cytokines (LIF, SDF-1, HGF, SCGF-β) that create a proinflammatory and immunosuppressive environment in the TME of breast cancer. The coloration of the dots serves to denote the secretion of cytokines, the green arrows indicating increases in expression and the red arrows indicating decreases.

Thus, heterotypic spheroid breast cancer models with CAFs demonstrate the formation of an aggressive and immunosuppressive tumor phenotype. The present data suggest that this phenotype is mediated by the coordinated action of key cytokines (LIF, SDF-1, HGF, and SCGF-β), which create a proinflammatory and immunosuppressive environment. It is proposed that the observed cytokine profile is characterized by a strategic shift from classical VEGF-dependent angiogenesis to a more complex mechanism aimed at active microenvironmental remodeling (Figure 9). In this model, SDF-1 and HGF promote the recruitment and activation of stromal cells, while LIF and, possibly, SCGF-β directly target tumor cells by activating key oncogenic signaling pathways such as JAK/STAT3 and PI3K/Akt. A significant consequence of the activation of these pathways is the direct and indirect induction of PD-L1 immune checkpoint expression. LIF, a known STAT3 activator, has been observed to bind directly to the PD-L1 gene promoter. In addition, other pathways (e.g., PI3K/Akt) have been shown to promote translational stabilization of its mRNA and protein. This synergistic effect ultimately leads to stable and elevated PD-L1 expression on the membranes of tumor and, likely, stromal cells, creating a powerful immune evasion mechanism that renders the tumor “invisible” to the immune system.

## Data Availability

The original contributions presented in this study are included in the article/Supplementary Material. Further inquiries can be directed to the corresponding author(s).

## Supplementary Materials

The following supporting information can be downloaded at: https://www.mdpi.com/article/10.3390/cells15020145/s1, Figure S1. Time-lapse transmitted light imaging formation of spheroid models BC. The data demonstrate the growth, stage, and time of spheroid formation from (a) tumor cells; (b) a mixture of tumor and endothelial cells; and (c) a mixture of tumor, endothelial and stromal cells; Figure S2. The characteristics of homotypic and heterotypic (tumor and endothelial cells) spheroids of breast cancer models. (a) Confocal microscopy of tumor cell-only (blue) and tumor-endothelial cell (blue and red) spheroids at day 5 after coculture. The scale bar is 100 µm. (b) Analysis of cell viability in a spheroid on day 5 of co-culture. Green signal (FDA)—live cells, red (PI)—dead cells, and endothelial cells (RFP), blue (Hoechst 33342)—total number of cells. The scale bar is 100 μm. (c) Formation of spheroids with the addition of 2.5% Matrigel^TM^. Confocal microscopy. Red signal indicating endothelial cells and green signal indicating tumor cells. The scale bar is 100 µm; Figure S3. The intercellular interactions in a heterotypic 3D cell model of breast cancer (3D-3). (a) MCF7; (b) MDA-MB-231; (c) SK-BR-3. Endothelial cells (red) form a network of interactions with each other, simulating an angiogenesis-like structure; stromal cells (green) are located near endothelial cell strands (f+end). Tumor cells (blue) form the periphery of the heterotypic 3D-model. Confocal microscopy. The scale bar is 100 µm; Figure S4. Characteristics of 2D cell cultures for constructing 3D models of BC. (a) Immunofluorescence staining of fibronectin (red signal) and DAPI nuclei (blue) in tumor and stromal cells. The scale bar is 100 µm. (b) Analysis of PD-L1-positive cells by flow cytometry. PD-L1—Programmed Death-Ligand 1. (c) Flow cytometry analysis of progenitor cell properties for surface expression of CD44 and CD24 of various breast cell lines. A subpopulation (CD44^+^/CD24^−^) of BC has been described as cancer stem cells. CD, cluster of differentiation; Figure S5. Migration and invasive activity profiling of cells. An analysis was conducted to ascertain the impact of migration at day 8 on a gelatin substrate, in conjunction with invasion at day 5 on a Matrigel^TM^ substrate; Figure S6. Tumor heterogeneity profiling and identification of cancer stem cells CSC in homotypic and heterotypic spheroids of BC models. Analysis of CD44 and CD24 by flow cytometry to identify CSCs, typically found as the CD44^+^/CD24^−^ population, which signifies aggressive, tumorigenic cells, while other combinations (CD44^+^/CD24^+^, CD24^+^/CD44^−^) mark different cell states, helping understand tumor heterogeneity, invasion, and therapy resistance; Figure S7. Spearman’s rank correlation of a 48-plex human cytokine array showing the relative fold change in cytokine/chemokine.

## Abbreviations

2D-models	two dimensions cellular culture
3D-models	three dimensions cellular culture
3D-2-models	three dimensions cellular culture from tumor and endothelial cells
3D-3-models	three dimensions cellular culture from tumor, endothelial and stromal cells
BC	breast cancer
BSA	bovine serum albumin
CAFs	cancer-associated fibroblasts
CSC	cancer stem cell
ECM	extracellular matrix
EGF	epidermal growth factor
FBS	fetal bovine serum
FDA	fluorescein diacetate
FGF	fibroblast growth factor
RFP	red fluorescent protein
SDF1	stromal cell-derived factor 1
VEGF	vascular endothelial growth factor
LIF	leukemia Inhibitory Factor
HGF	hepatocyte growth factor
PI	propidium iodide
SCGFβ	stem cell growth factor beta
TME	tumor microenvironment
