# Supplementary figures and images for: Heterotypic 3D Model of Breast Cancer Based on Tumor, Stromal and Endothelial Cells: Cytokines Interaction in the Tumor Microenvironment
Source: Cells. 2026 Jan 14;15(2):145. doi: 10.3390/cells15020145 (PMC12839793; doi:10.3390/cells15020145)

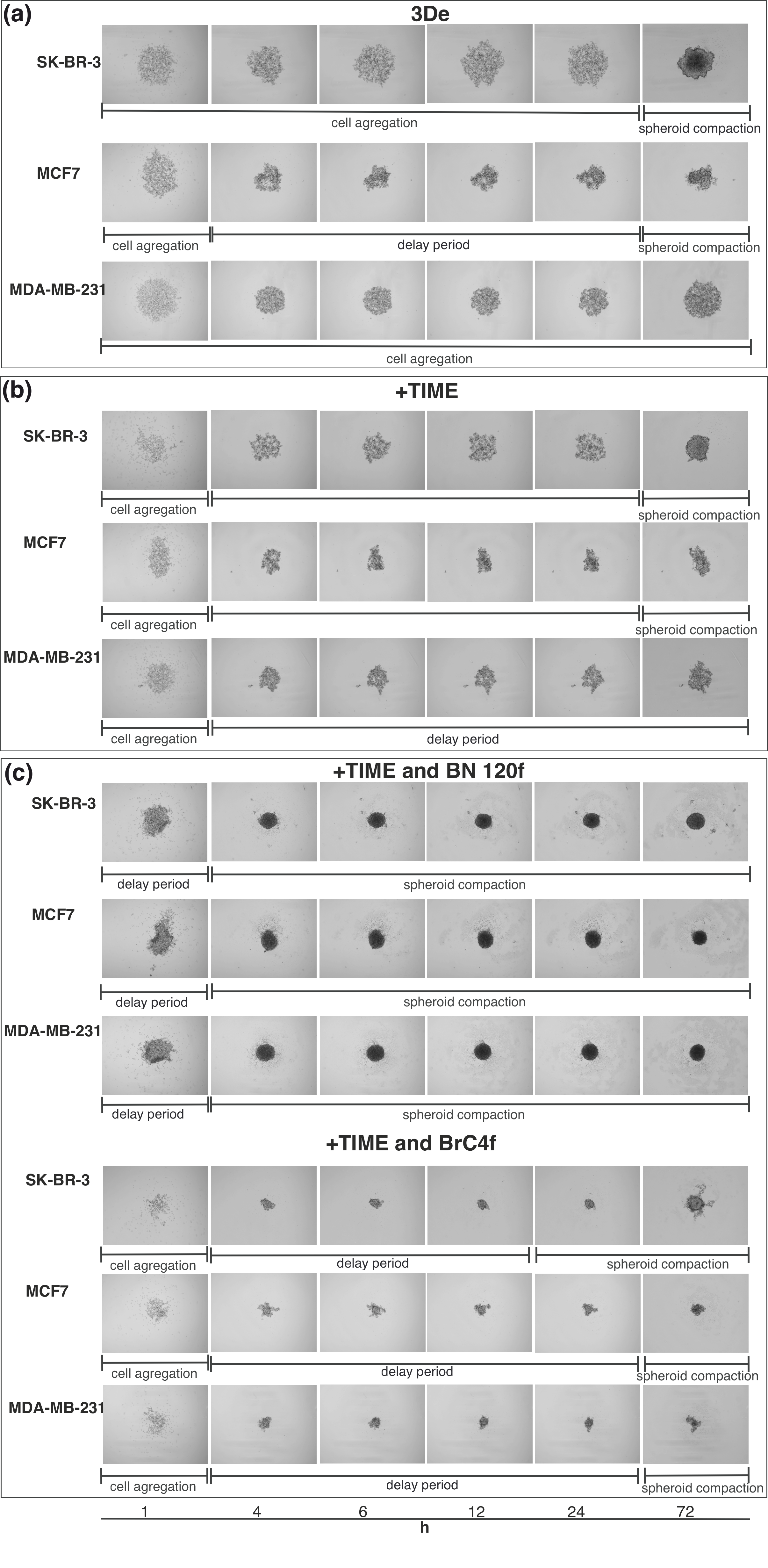

Supplement: Supplementary file 1 [file cells-15-00145-s001.zip › Supplementary/Figure_S1.png]

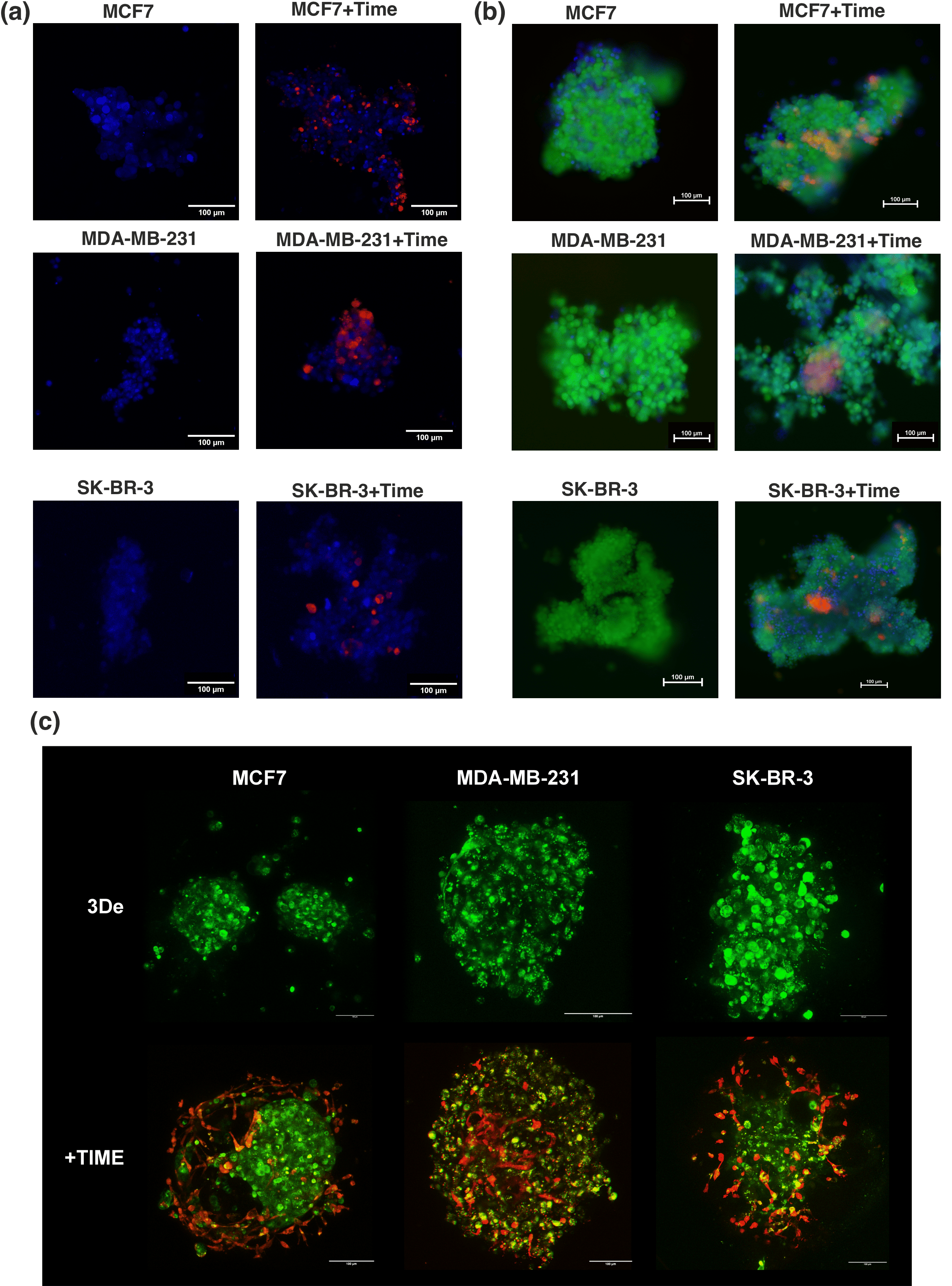

Supplement: Supplementary file 1 [file cells-15-00145-s001.zip › Supplementary/Figure_S2.png]

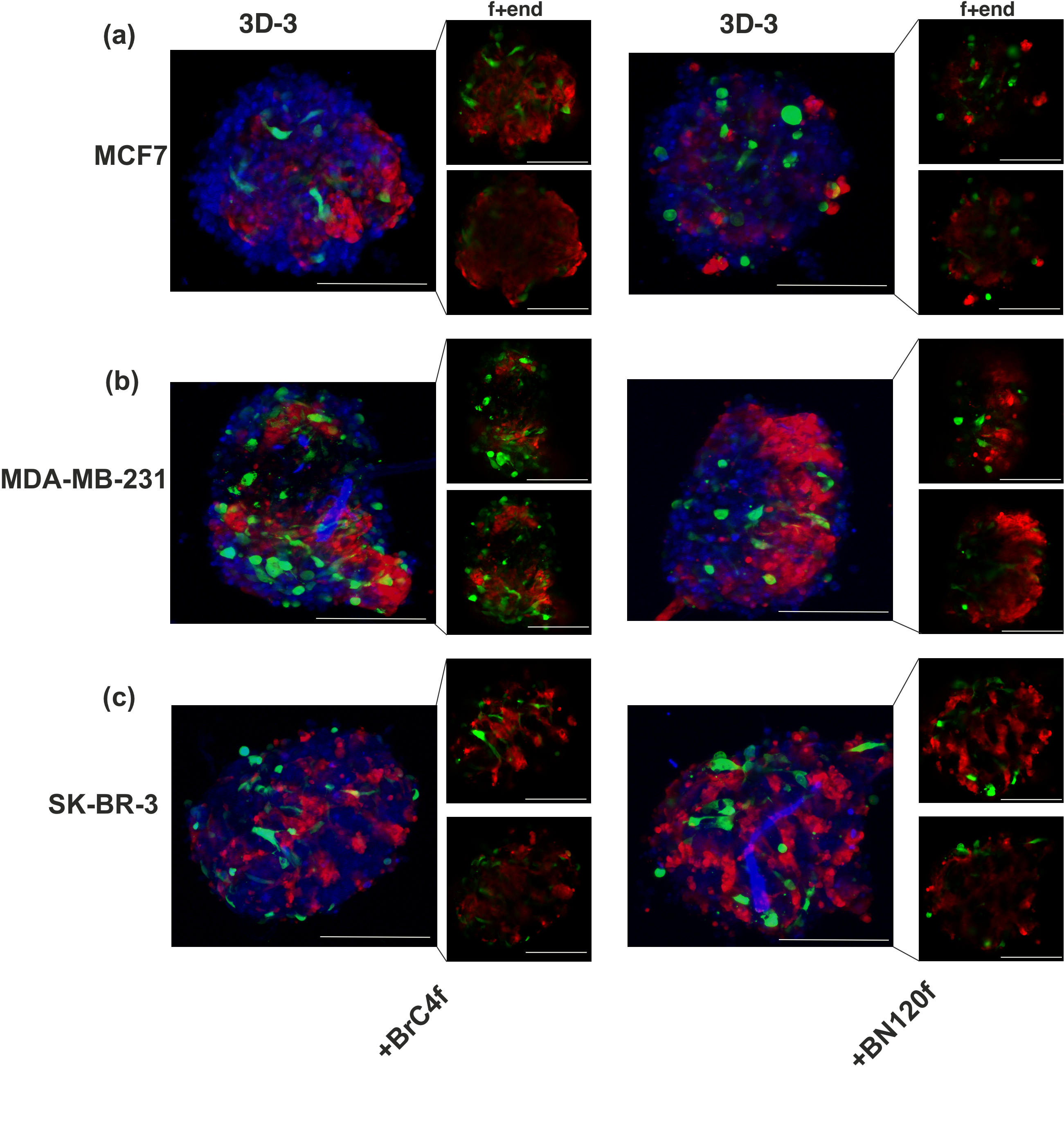

Supplement: Supplementary file 1 [file cells-15-00145-s001.zip › Supplementary/Figure_S3.png]

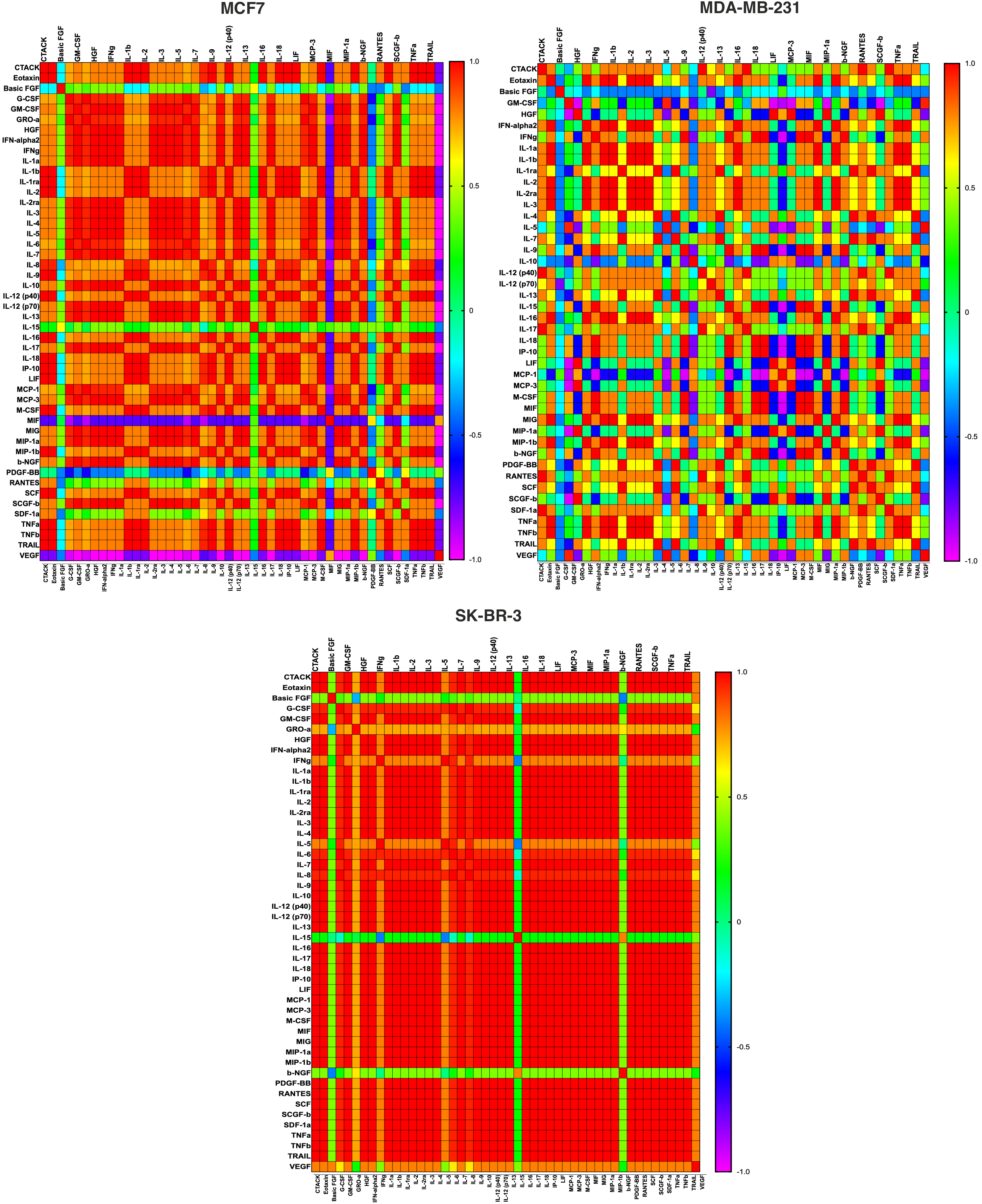

Supplement: Supplementary file 1 [file cells-15-00145-s001.zip › Supplementary/Figure_S7.png]

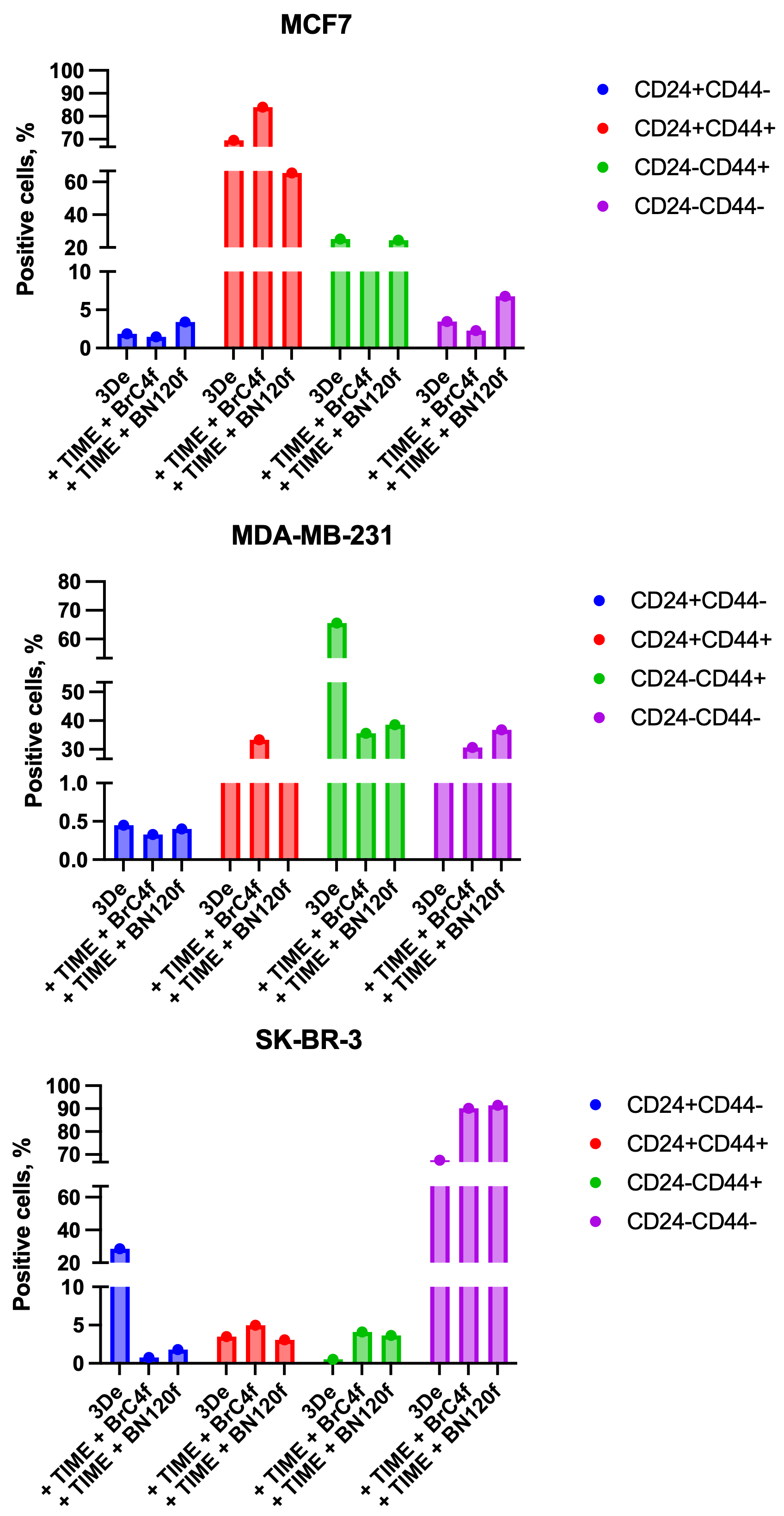

Supplement: Supplementary file 1 [file cells-15-00145-s001.zip › Supplementary/Figure_S6.png]

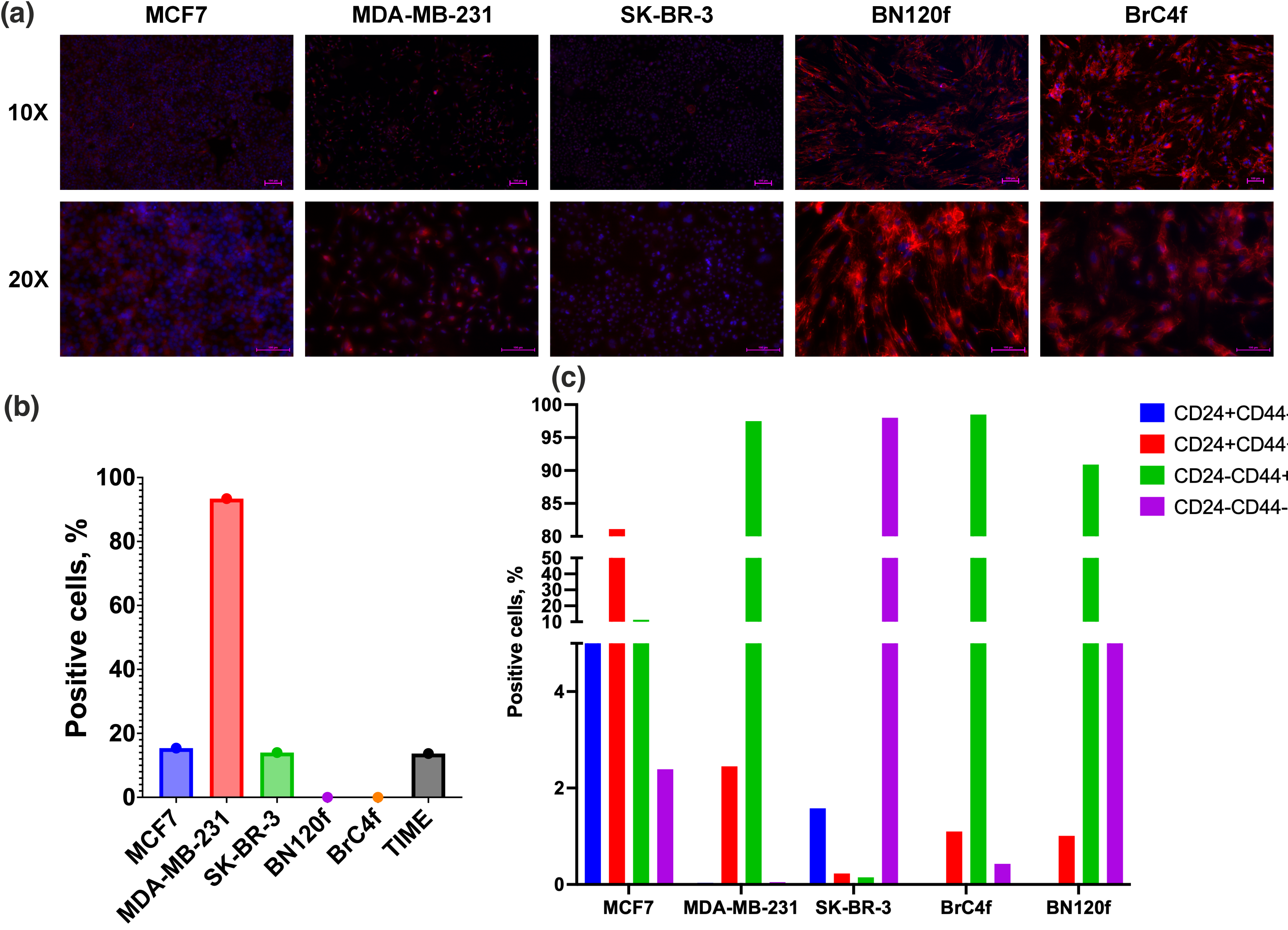

Supplement: Supplementary file 1 [file cells-15-00145-s001.zip › Supplementary/Figure_S4.png]

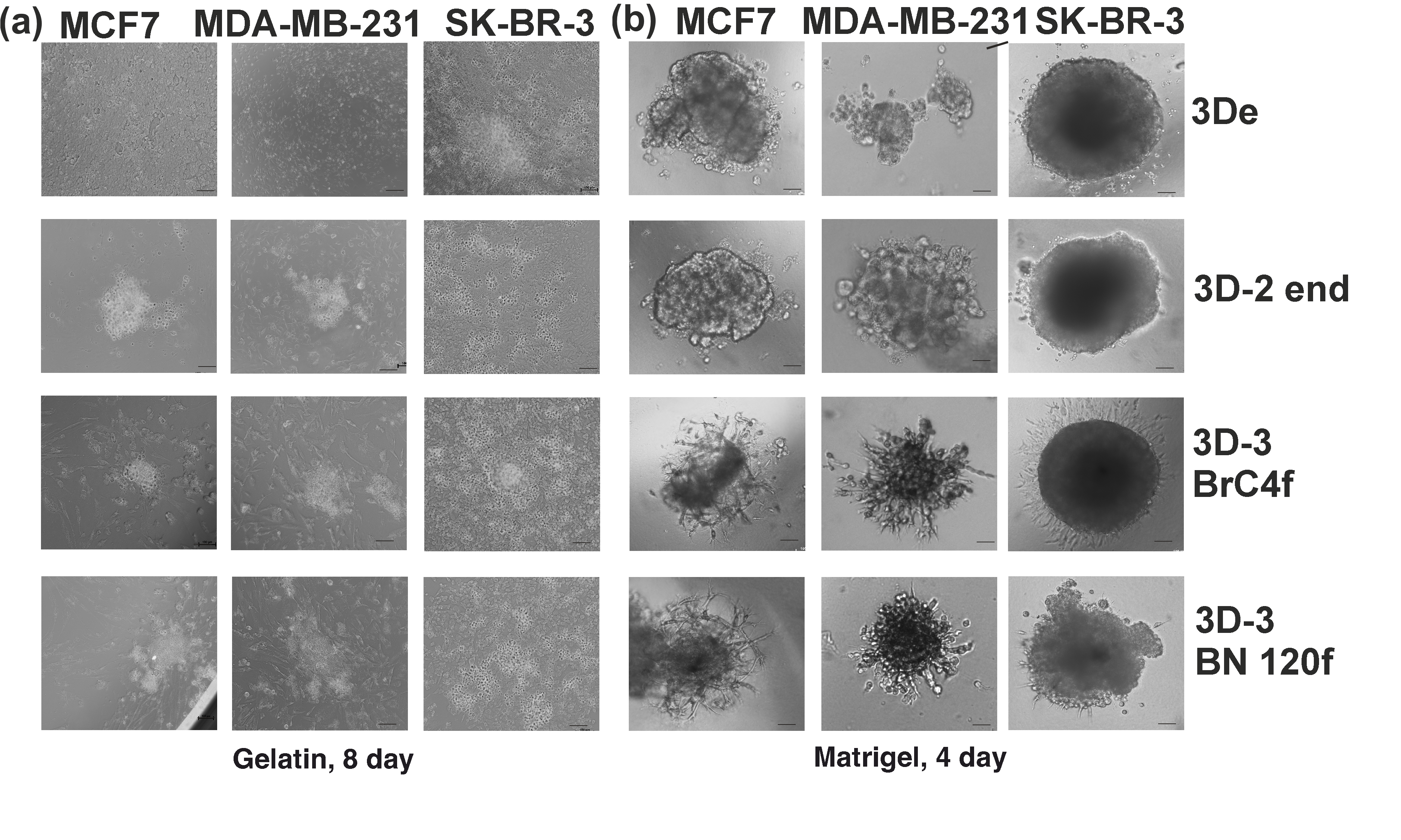

Supplement: Supplementary file 1 [file cells-15-00145-s001.zip › Supplementary/Figure_S5.png]
